# Supplementary material for: Non-conventional octameric structure of C-phycocyanin
Source: Commun Biol. 2021 Oct 29;4:1238. doi: 10.1038/s42003-021-02767-x (PMC8556327; doi:10.1038/s42003-021-02767-x)
Supplement: Supplementary file 2 — Supplementary information [file 42003_2021_2767_MOESM2_ESM.pdf]

Supplementary Information for

Non-conventional octameric structure of C-phycocyanin

Takuo Minato[1,2,3]*, Takamasa Teramoto[4]*, Naruhiko Adachi[5], Nguyen Khac Hung[1,2], Kaho Yamada[1,2], Masato Kawasaki[5,6], Masato Akutsu[5], Toshio Moriya[5], Toshiya Senda[5,6], Seiji Ogo[1,2,7], Yoshimitsu Kakuta[4,8]**, Ki-Seok Yoon[1,2,7]**

1 Department of Chemistry and Biochemistry, Graduate School of Engineering, Kyushu University, 744 Moto-oka, Nishi-ku, Fukuoka, 819-0395, Japan

2 International Institute for Carbon-Neutral Energy Research (WPI-I2CNER), Kyushu University, 744 Moto-oka, Nishi-ku, Fukuoka, 819-0395, Japan

3 Department of Applied Chemistry, Graduate School of Advanced Science and Engineering, Hiroshima University, 1-4-1 Kagamiyama, Higashi-Hiroshima, Hiroshima, 739-8527, Japan

4 Department of Bioscience and Biotechnology, Faculty of Agriculture, Kyushu University, 744 Moto-oka, Nishi-ku, Fukuoka, 819-0395, Japan

5 Structural Biology Research Center, Institute of Materials Structure Science, High Energy Accelerator Research Organization (KEK), 1-1 Oho, Tsukuba, Ibaraki, 305-0801, Japan

6 Department of Materials Structure Science, School of High Energy Accelerator Science, The Graduate University of Advanced Studies (Soken-dai), 1-1 Oho, Tsukuba, Ibaraki 305-0801, Japan.

7 Center for Small Molecule Energy, Kyushu University, 744 Moto-oka, Nishi-ku, Fukuoka, 819-0395, Japan

8 Laboratory of Structural Biology, Graduate School of System Life Sciences, Kyushu University, 744 Moto-oka, Nishi-ku, Fukuoka, 819-0395, Japan

** corresponding authors

Y. Kakuta, Department of Bioscience and Biotechnology, Faculty of Agriculture, Kyushu University, 744 Moto-oka, Nishi-ku, Fukuoka, 819-0395, Japan

Tel: +81-92-802-4709

E-mail: kakuta@agr.kyushu-u.ac.jp

K.-S. Yoon, International Institute for Carbon-Neutral Energy Research (WPI-I2CNER), Kyushu University, 744 Moto-oka, Nishi-ku, Fukuoka 819-0395, Japan

Tel: +81-92-802-6688

E-mail: yoon@i2cner.kyushu-u.ac.jp

* These authors contributed equally to this work.

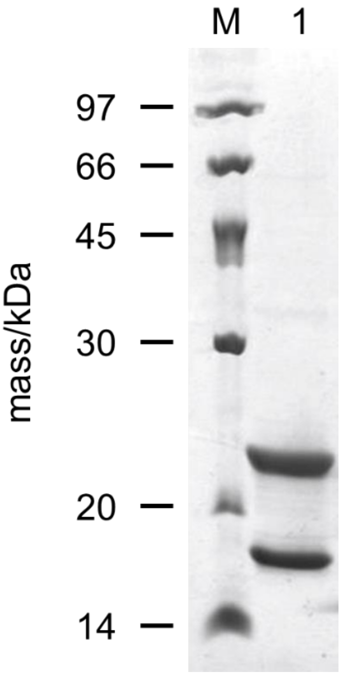

Supplementary Fig. 1 SDS-PAGE of *Tl*CPC. Lane 1, *Tl*CPC in 10 mM potassium phosphate buffer solution (pH 7.0). M, a marker.

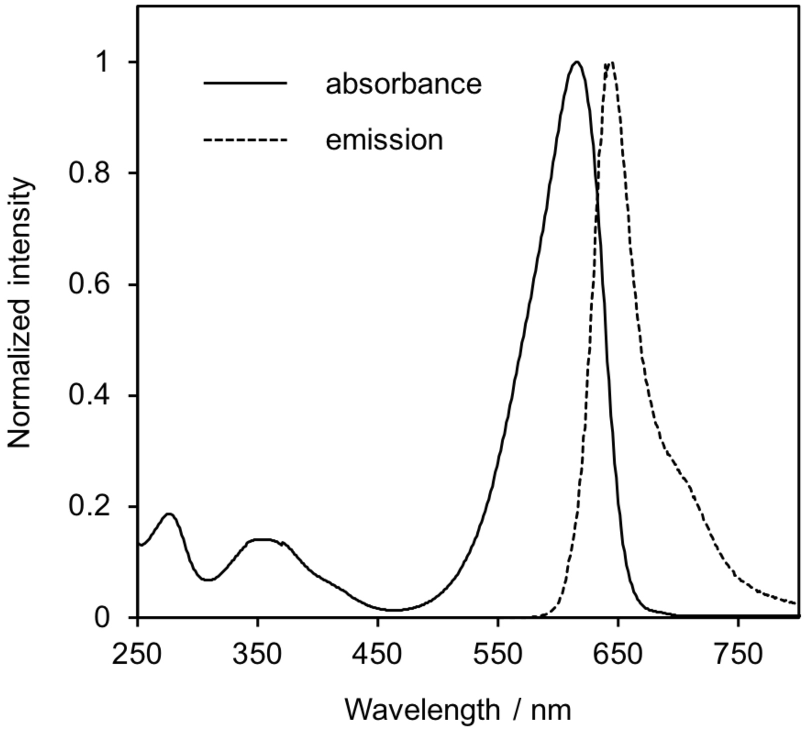

Supplementary Fig. 2 UV-vis and fluorescence spectra of *Tl*CPC. UV-vis spectrum of *Tl*CPC (0.17 mg/mL) was measured in 10 mM potassium phosphate buffer solution (pH 7.0). Fluorescence spectrum was measured with the excitation wavelength at 436 nm.

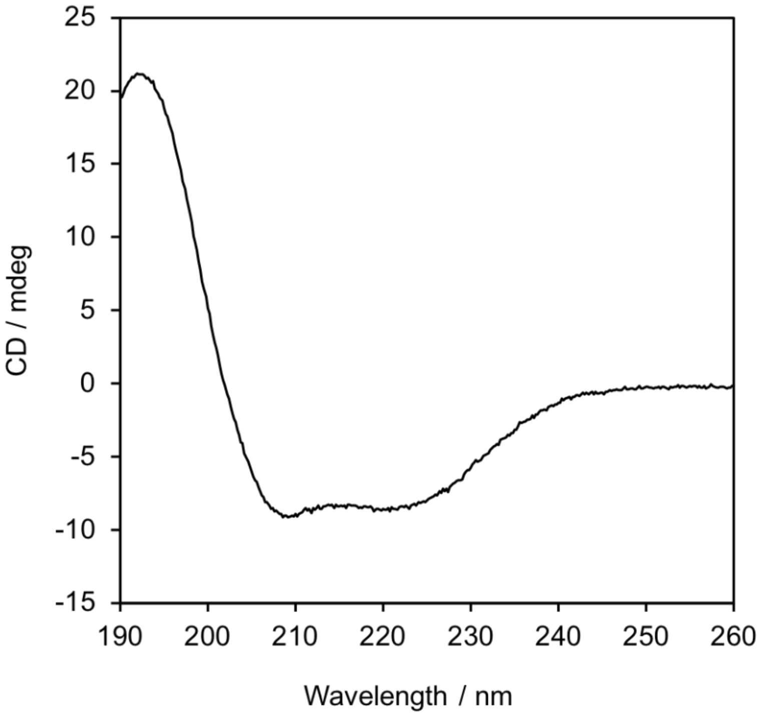

Supplementary Fig. 3 CD spectrum of *Tl*CPC. CD spectrum of *Tl*CPC (56 μg /mL) was measured in 10 mM potassium phosphate buffer solution (pH 7.0).

α subunit

```
                          helix A            helix B             helix C       helix D        helix E
TlCPC          1    M-KTPITEAIAAADTQGRFLSNTELQAVNGRFERAAASMEAARALTNNAQQLIDGAANAVYQKFPYTTQMQGANFASDSR  79
TvCPC          1    M-KTPITEAIAAADTQGRFLSNTELQAVDGRFKRAVASMEAARALTNNAQSLIDGAAQAVYQKFPYTTTMQGSQYASTPE  79
TeCPC          1    M-KTPITEAIAAADTQGRFLSNTELQAVDGRFKRAVASMEAARALTNNAQSLIDGAAQAVYQKFPYTTTMQGSQYASTPE  79
SeCPC          1    MSKTPLTEAVAAADSQGRFLSSTELQVAFGRFRQAASGLAAAKALANNADSLVNGAANAVYSKFPYTTSTPGNNFASTPE  80
SPCC6803CPC    1    M-KTPLTEAVSTADSQGRFLSSTELQIAFGRLRQANAGLQAAKALTDNAQSLVNGAAQAVYNKFPYTTQTQGNNFAADQR  79
LN62DMCPC      1    M-KTPLTEAVSVADSQGRFLSSTEIQVAFGRFRQAKAGLEAAKALTSKADSLISGAAQAVYNKFPYTTQMQGPNYAADQR  79
P1w0831CPC     1    M-KTPLTEAVSAADSQGRFLSTTETQVAFGRFRQATSGLAAAKALSEKAESLASGAANAVYSKFPYTTSMTGANYASSQT  79
AmCPC          1    M-QTPLIEAVSSADSQGRFLSSTELQVAFGRFRQAAASLDAAKTLNSKADSLADGAANAVYQKFPYTTQMTGSNYASTPE  79
ApCPC          1    M-KTPLTEAVSVADSQGRFLSSTEIQVAFGRFRQAKAGLEAAKALTSKADSLISGAAQAVYNKFPYTTQMQGPNYAADQR  79
PrCPC          1    M-KTPLTEAVAVADSQGRFLSSTEVQTAFGRFRQAGASLAAAKGLTEKASSLTSGAAQAVYNKFPYTTQMQGPNYAATQT  79
MdCPC          1    M-KTPLTEAVAAADSQGRFLSSTEIQTAFGRFRQASASLAAAKALTEKASSLASGAANAVYSKFPYTTSQNGPNFASTQT  79
NWR13CPC       1    M-KTPLTEAVATADSQGRFLSSTELQVAFGRFRQATASLDAAKGLSSKAQSLADGAANAVYQKFPYTTQMQGNNFASTPT  79
NR76DMCPC      1    -KKTPLTEAISAADSQGRYLSSTEIQVAFGRFRQAPASLQAAKSLSANASRLTEGAAQAVYNKFPYTTQQQGPNFAYDQR  79
GvCPC          1    M-KTVITEVIASADSQGRFLNNTELQAANGRFQRATASMEAARALTSNADSLVKGAVQEVYNKFPYLTQPGQMGYGDTNQ  79
GsCPC          1    M-KTPITEAIAAADNQGRFLSNTELQAVNGRYQRAAASLEAARSLTSNAQRLINGAAQAVYSKFPYTSQMPGPQYASSAV  79
GcCPC          1    M-KTPITEAIASADSQGRFLSNGELQSINGRYQRATASLEAARSLTSNAERLISGAAQSVYSKFPYTTQMQGPNYAADAT  79
```

```
                          helix E          helix F   helix G     helix H                helix I
TlCPC         80    GKSKCARDIGYYLRIITYSLVAGGTGPLDEYLIAGLDEINRTFDLSPSWYVEALKYIKAN--HGLSGQAANEANTYIDYAINALS  162
TvCPC         80    GKAKCARDIGYYLRMITYCLVAGGTGPMDEYLIAGLSEINSTFDLSPSWYIEALKYIKAN--HGLTGQAAVEANAYIDYAINALS  162
TeCPC         80    GKAKCARDIGYYLRMVTYCLVAGGTGPMDEYLIAGLSEINSTFDLSPSWYIEALKYIKAN--HGLTGQAAVEANAYIDYAINALS  162
SeCPC         81    GKAKCARDIGYYLRIVTYALVAGGTGPIDEYLLAGLDEINKTFDLAPSWYVEALKYIKAN--HGLSGDSRDEANSYIDYLINALS  163
SPCC6803CPC   80    GKDKCARDIGYYLRIVTYCLVAGGTGPLDEYLIAGIDEINRTFDLSPSWYVEALKYIKAN--HGLSGDARDEANSYLDYAINALS  162
LN62DMCPC     80    GKDKCARDIGYYLRMVTYCLIAGGTGPMDEYLIAGIDEINRTFELSPSWYIEALKYIKAN--HGLSGDAAVEANSYLDYAINALS  162
P1w0831CPC    80    GKDKCVRDIGYYIRMVTYCCVVGGTGPMDDYLVAGIAEINRTFDLSPSWYVEALKYVKAN--HGLSGDSAVEANSYIDYAINALS  162
AmCPC         80    GKAKCVRDIGYYLRIISYCLIAGGTGPLDDYLINGLAEINRTFDLSPSWYVEALKHIKAN--HGLSGDSAVEANSYIDYAINALS  162
ApCPC         80    GKDKCARDIGYYLRMVTYCLIAGGTGPMDEYLIAGIDEINRTFELSPSWYIEALKYIKAN--HGLSGDAAVEANSYLDYAINALS  162
PrCPC         80    GKDKCVRDIGYYLRMVTYCLVVGGTGPMDDYLIGGLAEINKTFELSPSWYIEALKYIKAN--HGLSGDPAVEANSYLDYAINALS  162
MdCPC         80    GKDKCVRDIGYYLRMVTYCLVVGGTGPLDDYLIGGIAEINRTFDLSPSWYVEALKYIKAN--HGLSGDPAVEANSYIDYAINALS  162
NWR13CPC      80    GKAKCARDIGYYIRIITYCLIAGGTGPLDDYLIGGLAEINRTFDLSPSWYVEALKYIKAN--HGLSGDPAVEANSYIDYAINALS  162
NR76DMCPC     80    GKAKCVRDVGYYLRIITYALVVGGTGPLDDYLISGLAEINKTFDLSPSWYIEALKYIKAN--HGLSGDPAVEANSYIDYVINTLS  162
GvCPC         80    --AKCARDISHYLRFITYSLVAGGTGPLDDYIVAGLREVNRTFNLSPSWYIEALKHIKGKVGSQLSGQPLTEANAYIDYCINALS  162
GsCPC         80    GKAKCARDIGYYLRMVTYCLVVGGTGPMDEYLIAGLEEINRTFDLSPSWYVEALNYVKSN--HGLSGQAANEANTYIDYAINALS  162
GcCPC         80    GKAKCARDIGYYLRMVTYCLVVGATGPMDEYLIAGLSEINRSFELSPSWYIEALEYIKDS--HALSGQAANEANTYLDYAINALS  162
```

β subunit

```
                          helix A        helix B             helix C              helix D      helix E
TlCPC          1    M-LDAFAKVVSQADTKGEFLSSAQLDALSNVVKDGSKRLDAVNRMTSNASTIVANAARSLFEEQPQLIQPGGNAYTNRRM  79
TvCPC          1    M-LDAFAKVVAQADARGEFLTNAQFDALSNLVKEGNKRLDAVNRITSNASTIVANAARALFAEQPQLIQPGGNAYTNRRM  79
TeCPC          1    M-LDAFAKVVAQADARGEFLTNAQFDALSNLVKEGNKRLDAVNRITSNASTIVANAARALFAEQPQLIQPGGNAYTNRRM  79
SeCPC          1    MTFDAFTKVVAQADARGEFLSDAQLDALSRLVAEGNKRIDTVNRITGNASSIVANAARALFAEQPSLIAPGGNAYTNRRM  80
SPCC6803CPC    1    M-FDVFTRVVSQADARGEYLSGSQLDALSATVAEGNKRIDSVNRITGNASAIVSNAARALFAEQPQLIQPGGNAYTSRRM  79
LN62DMCPC      1    M-FDAFTKVVSQADTRGEMLSTAQIDALSQMVAESNKRLDVVNRITSNASTIVSNAARSLFAEQPQLIAPGGNAYTSTRM  79
P1w0831CPC     1    M-YDAFAKVVSQADSRGAYISASQIDALSAMVADGSKRLDAVNRITSNSSAIVANAARALFAEQPALIAPGGNAYTSRRM  79
AmCPC          1    M-LDAFTKVVSQADTRGAYVSDAEVDALKAMVADANKRIDAVNRITGNASTIVANAARALFADQPQLCAPGGNAYTSRRM  79
ApCPC          1    M-FDAFTKVVSQADTRGEMLSTAQIDALSQMVAESNKRLDVVNRITSNASTIVSNAARSLFAEQPQLIAPGGNAYTSRRM  79
PrCPC          1    M-FDAFTKVVSQADARGAYLTTDQIDALTALVSDGNKRMDVVNRITSNSSKIVADAARSLFAEQPQLIAPGGNAYTSRRM  79
MdCPC          1    M-LDAFAKVVSQADARGEYLSGSQIDALSALVADGNKRMDVVNRITGNSSTIVANAARSLFAEQPQLIAPGGNAYTSRRM  79
NWR13CPC       1    M-LDAFTKVVSQADTRGAYVSDAEIDALKEMVAAGAKRMDVVNRITGNASTIVANAARGLFAEQPQLIAPGGNAYTNRRM  79
NR76DMCPC      1    M-LDAFAKVVAQADTRGEYLTDAQLDALSELVKEGNKRADAVNRITSNASAIVAAAVRDLWAEQPQLIAPGGNAYTSRRA  79
GvCPC          1    M-QDAFTKAIVAADLRGSFLSEQELNQLTNLVKESNKRLDAVNAITGNAAEIISDAAHKLFAEQTDLIRPGGNAYPNRRM  79
GsCPC          1    M-LDAFAKVVAQADARGEFLSNTQLDALSKMVSEGNKRLDVVNRITSNASAIVTNAARALFSEQPQLIQPGGNAYTNRRM  79
GcCPC          1    M-LDAFAKVVAQADARGEFLSNTQLDALANMIAEGNKRLDIVNRINSNASAIVSNSARALFAEQPQLIQPGGNAYTNRRM  79
```

```
                          helix E         helix F  helix G        helix H                          helix I
TlCPC         80    AACLRDMEIILRYVTYATLAGDSSVLDDRCLNGLRETYQALGVPGGSVAAGVAKMKDAAIAIVNDPNGITKGDCSALVSEIASYFDRAAAAVA  172
TvCPC         80    AACLRDMEIILRYVTYAILAGDSSVLDDRCLNGLRETYQALGTPGSSVAVAIQKMKDAAIAIANDPNGITPGDCSALMSEIAGYFDRAAAAVA  172
TeCPC         80    AACLRDMEIILRYVTYAILAGDSSVLDDRCLNGLRETYQALGTPGSSVAVAIQKMKDAAIAIANDPNGITPGDCSALMSEIAGYFDRAAAAVA  172
SeCPC         81    AACLRDMEIILRYVTYAVFTGDASILDDRCLNGLRETYLALGVPGASVAEGVRKMKDAAVAIVSDRNGITQGDCSAIISELGSYFDKAAAAVA  173
SPCC6803CPC   80    AACLRDMEIILRYVTYATFTGDASVLEDRCLNGLRETYVALGVPGASVAAGVQKMKEAALDIVNDPNGITRGDCSAIVAEIAGYFDRAAAAVA  172
LN62DMCPC     80    AACLRDMEIILRYVTYAVFAGDASVLEDRCLNGLRETYLALGTPGSSVAVGVGKMKEAALAIVNDPAGITPGDCSALASEIASYFDRACAAVS  172
P1w0831CPC    80    AACLRDMEIVLRYVTYAIYSGDASILEDRCLNGLKETYLALGTPGSSVAVGIGKMKDAAIAIANDPNGVTRGDCSALMSEIGSYFDKAAAAVA  172
AmCPC         80    AACLRDMEIILRYVTYAVYTGDASVLNDRCLNGLRETYSALGVPGGSVAAGVQKMKEAAIEIANDPKGITQGDCSNLMAEIGSYFDLASSAVG  172
ApCPC         80    AACLRDMEIILRYVTYAVFAGDASVLEDRCLNGLRETYLALGTPGSSVAVGVGKMKEAALAIVNDPAGITPGDCSALASEIAGYFDRAAAAVS  172
PrCPC         80    AACLRDMEIILRYVTYAIFSGDASVLDDRCLNGLRETYIALGTPGASVAVGVEKMKQAALAIAGDTSGITPGDCASIMSEVASYFDRAAAAVA  172
MdCPC         80    AACLRDMEIILRYVTYAIFAGDASVLDDRCLNGLKETYLALGTPGSSVAVGVQKMKDAALAIAGDTNGITRGDCASLMAEVASYFDKAASAVA  172
NWR13CPC      80    AACLRDMEIILRYVTYAVFAGDASVLDDRCLNGLRETYQALGVPGASVATGVSKMKEAAIAIANDPNGVTQGDCSSLMAELGSYFDRAAAAVS  172
NR76DMCPC     80    AACIRDLDIILRYVTYAIFAGDSSVLDDRALNGLRETYLALGTPGASVAVGIQKLKDASLKIVNDPNGITRGDCSALAAEIASYFDRAAAAVA  172
GvCPC         80    AACLRDMEIILRYVSYALLAGDASVLEDRCLNGLKETYVALGTPTRSVARAVQLMKETAIGYVNSPSGVTRGDCSALVNEAATYFDKAAASIA  172
GsCPC         80    AACLRDMEIILRYVSYAIIAGDSSVLDDRCLNGLRETYQALGVPGASVAVGVEKMKDSAIAIANDPSGITTGDCSALMAEVGTYFDRAATAVQ  172
GcCPC         80    AACLRDMEIVLRYVSYAEIAGDSSVLDDRCLNGLRETYQALGTPGSSVAVAIEKMKEASVSDANDSSGTPSGDCSSLSAELGTYFDRAASAVS  172
```

Supplementary Fig. 4 Multiple sequence alignment for structurally characterized CPCs. CPCs from *T. vulcanus* (*Tv*CPC; PDB ID, 1I7Y), *T. elongatus* BP-1 (*Te*CPC; PDB ID, 3L0F), *Synechococcus*

elongatus PCC 7942 (*Se*CPC; PDB ID, 4H0M), *Synechocystis* sp. PCC 6803 ($S_{PCC6803}$CPC; PDB ID, 4F0T), *Leptolyngbya* sp. N62DM (L_{N62DM}CPC; PDB ID, 4L1E), *Pseudanabaena* sp. lw0831 (P_{lw0831}CPC; PDB ID, 5TOU), *Acaryochloris marina* (*Am*CPC; PDB ID, 5OOK), *Arthrospira platensis* (*Ap*CPC; PDB ID, 1GH0), *Phormidium rubidium* (*Pr*CPC; PDB ID, 4YJJ), *Microchaete diplosiphon* (*Md*CPC; PDB ID, 1CPC), *Nostoc* sp. WR13 (N_{WR13}CPC; PDB ID, 6HRN), *Nostoc* sp. R76DM (N_{R76DM}CPC; PDB ID, 6JPR), *Gloeobacter violaceus* (*Gv*CPC; PDB ID, 2VJR), *Galdieria sulphuraria* (*Gs*CPC; PDB ID, 3BRP), and *Gracilaria chilense* (*Gc*CPC; PDB ID, 2BV8). Conserved Cys residue at PCBs and methylated Asn residue are highlighted in yellow and blue, respectively.

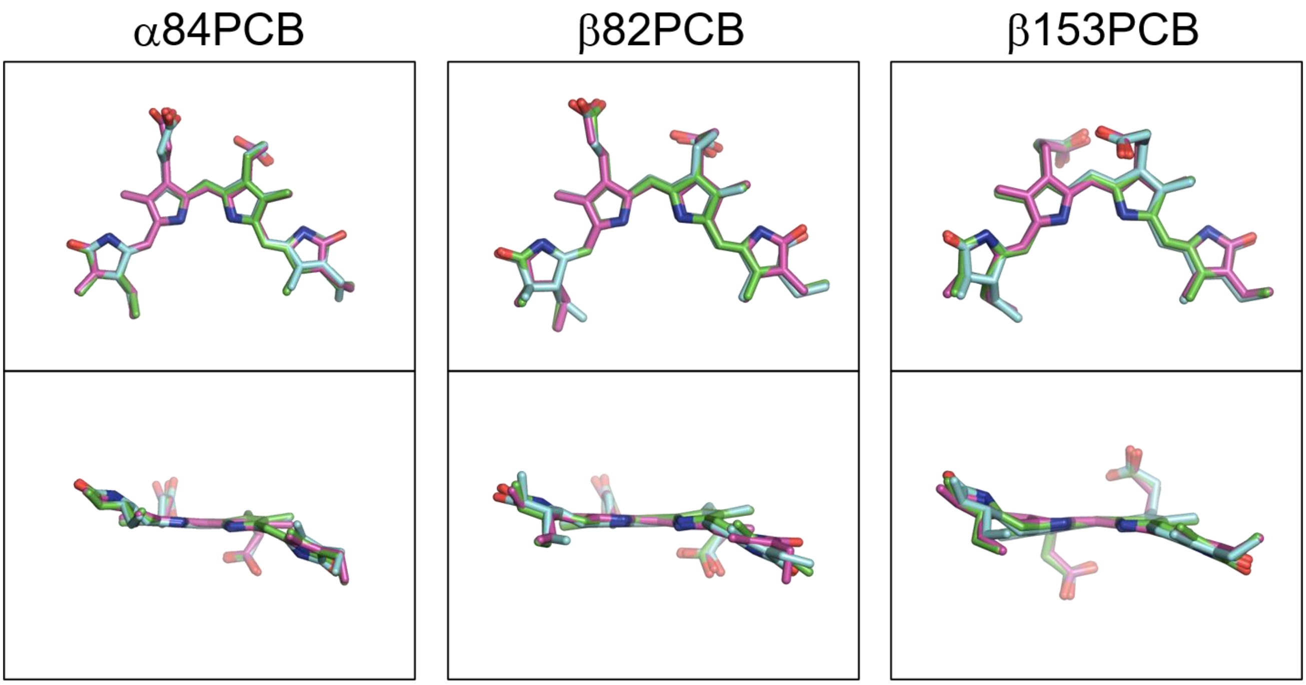

Supplementary Fig. 5 PCBs in crystal models of *Tl*CPC. The superimposition of the PCB structures from *Tl*CPC-6 (green), *Tl*CPC-8 (cyan), and *Se*CPC (magenta). The top and bottom panels show the top and side views of PCBs, respectively.

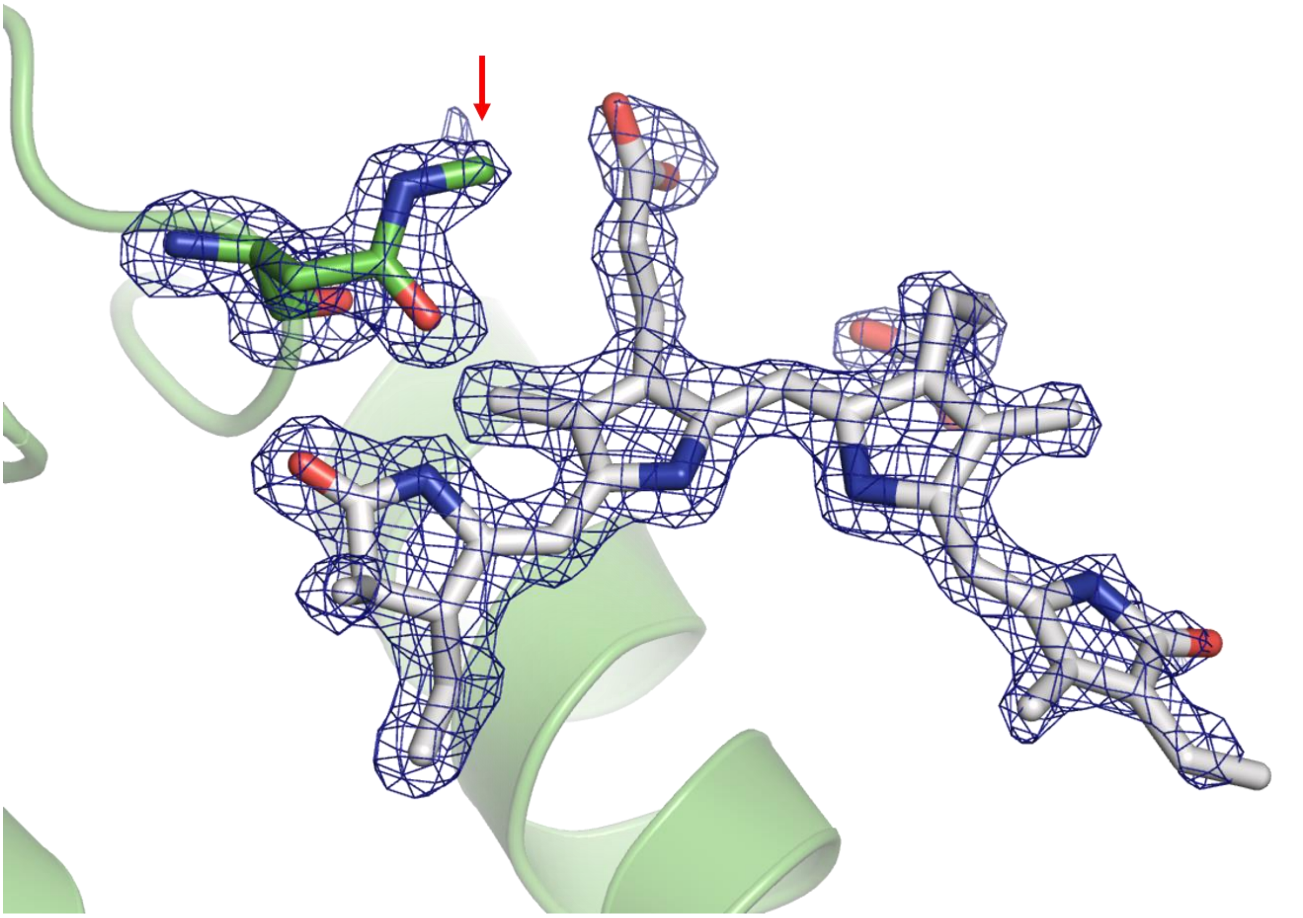

Supplementary Fig. 6 Methylation of βAsn72 in *Tl*CPC-6. Electron density maps of methylated βAsn72 and β83PCB in *Tl*CPC-6. F_o–F_c omit electron density maps contoured at 4.5σ are superimposed on βAsn72 and β83PCB. Red arrow indicates the methylation moiety of βAsn72.

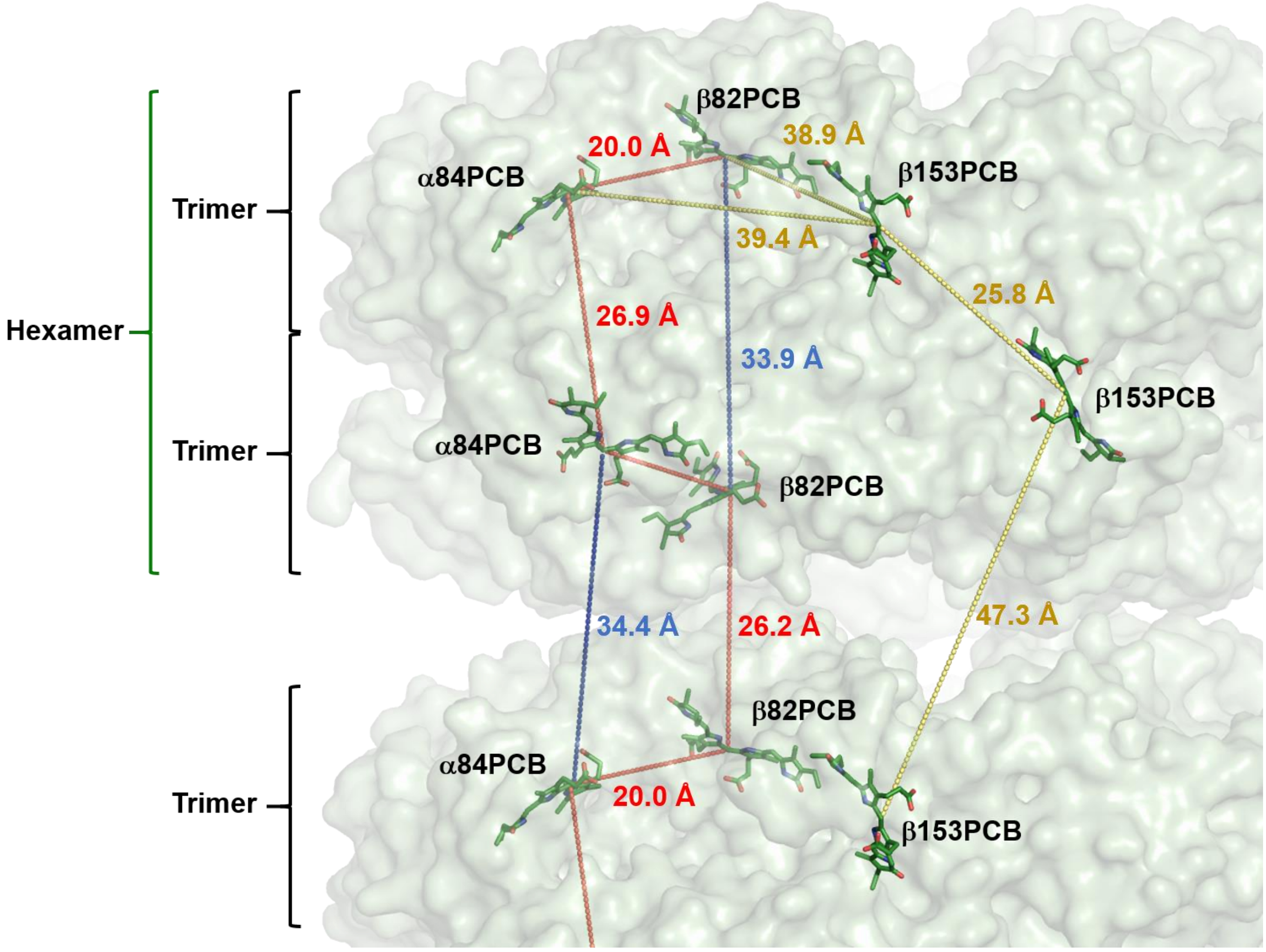

Supplementary Fig. 7 Intra- and inter-protein PCB distances of *Tl*CPC-6. PCBs in *Tl*CPC-6 are represented by green sticks. CPCs are shown in green surface representation with 70% transparency. The proposed energy transfer pathway (the shortest distances between PCBs) is shown in red. The next shortest distances are colored in blue. Distances involved in β153PCB are colored in yellow.

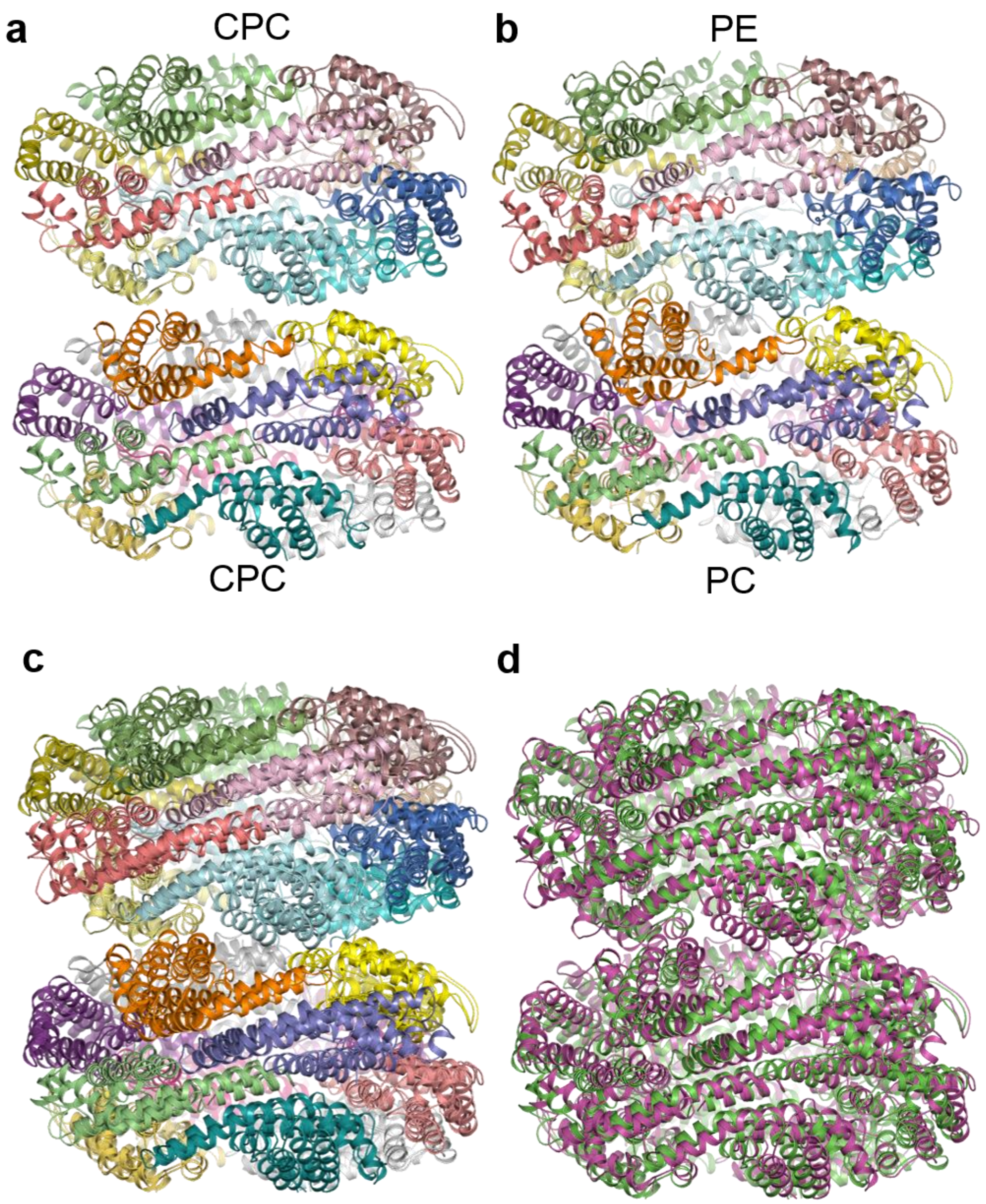

Supplementary Fig. 8 Superimposition of double-layered [(αβ)$_6$]$_2$ structures. a Crystal model of double-layered CPC–CPC structure of *Tl*CPC-6. **b** Cryo-EM model of double-layered PE–PC structure of PBS from *P. purpureum*. Positionally equivalent subunits in CPC-CPC and PE-PC structures are shown at same color. **c,d** Superimpositions of the CPC-CPC structure from *Tl*CPC-6 crystal packing and the PE-PC structure from *Pp*PBS cryo-EM structure. Each subunit is shown by cartoon representation with different color in **a–c**, whereas CPC–CPC structure of *Tl*CPC-6 and PE–PC structure of PBS are represented by green and magenta, respectively, in **d**.

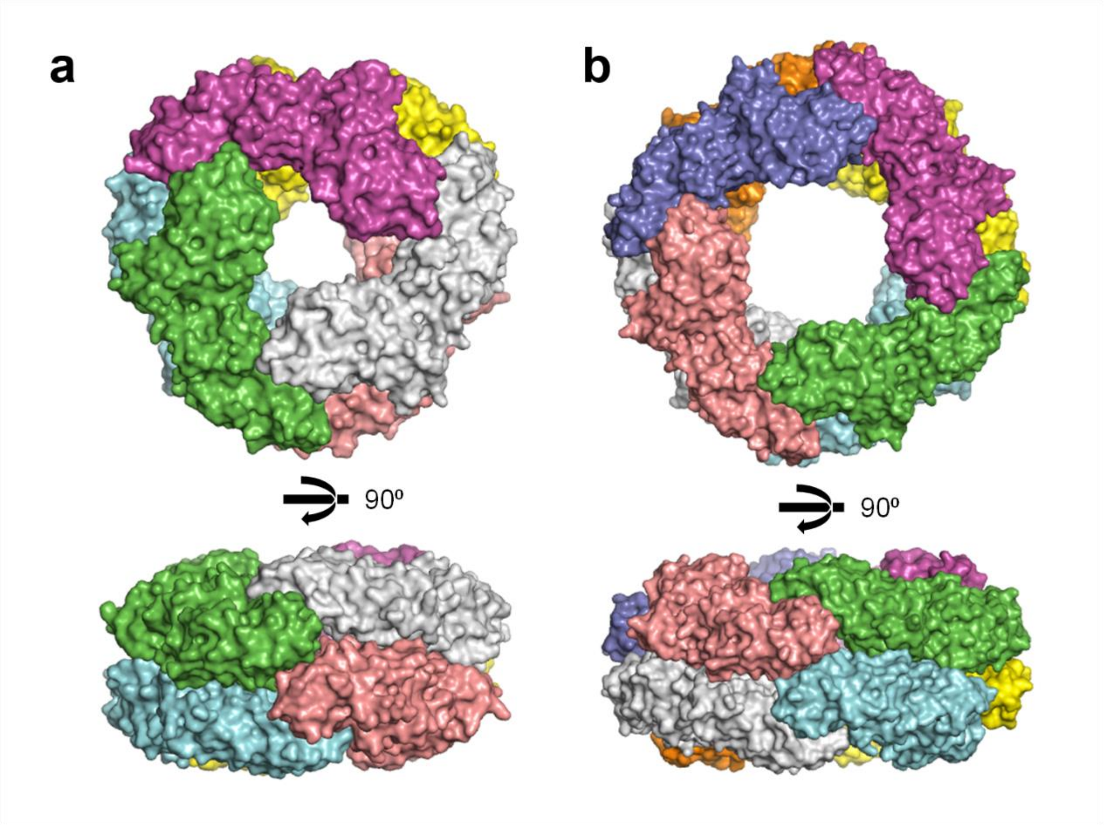

Supplementary Fig. 9 Monomer interfaces of *Tl*CPC-6 and *Tl*CPC-8. Assemblies of monomers (αβ) in *Tl*CPC-6 (**a**) and *Tl*CPC-8 (**b**). Each monomer is shown by surface representation with different color.

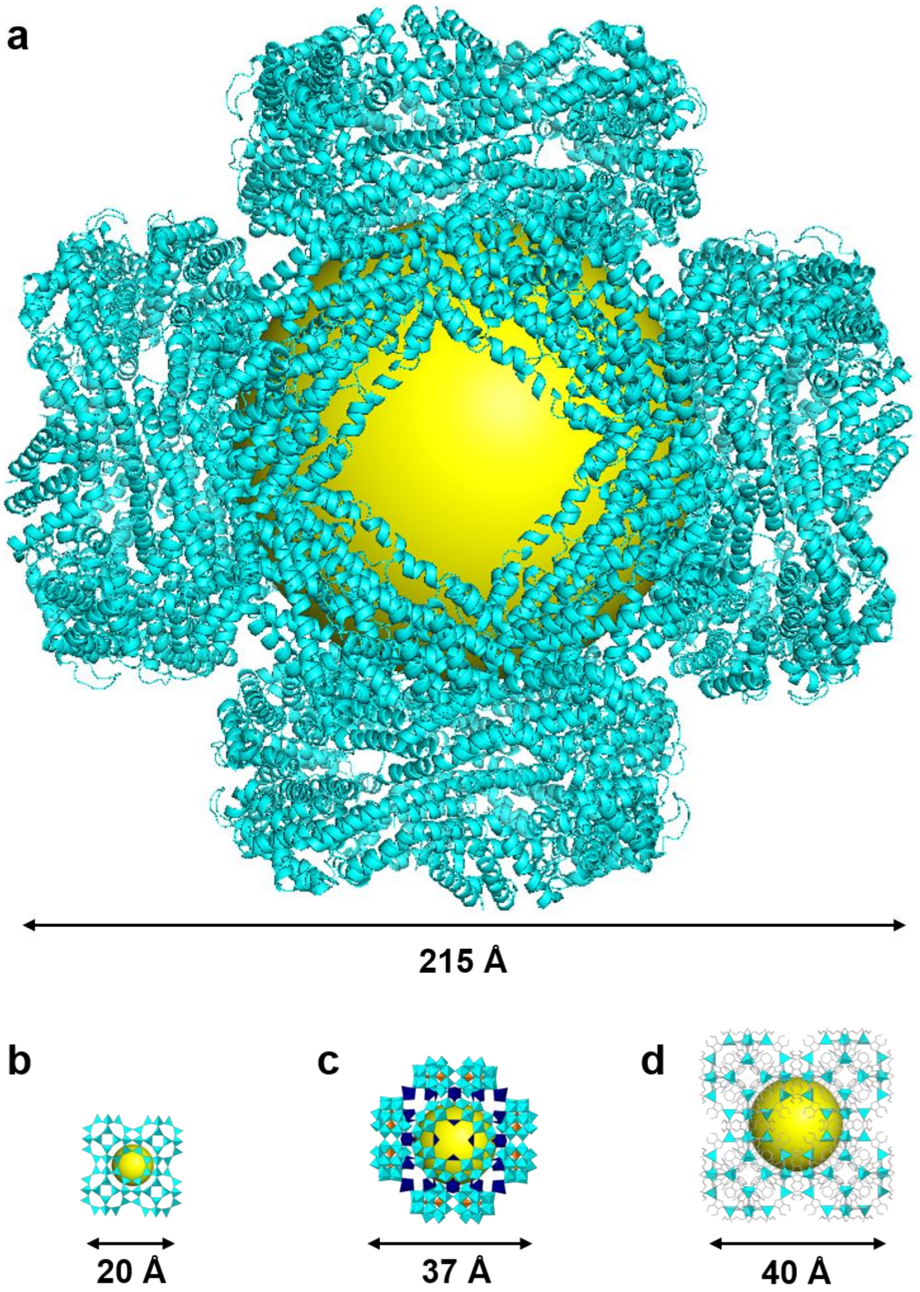

Supplementary Fig. 10 Void spaces in zeolitic crystal structures. Crystal structures of **a** *Tl*CPC-8, **b** zeolite A, **c** polyoxometalate ($K_{18}Li_6[Mn_8(H_2O)_{48}P_8W_{48}O_{184}]\cdot 108H_2O$), and **d** metal-organic framework (ZIF-20). Void spaces located in the cavities are represented by yellow spheres.

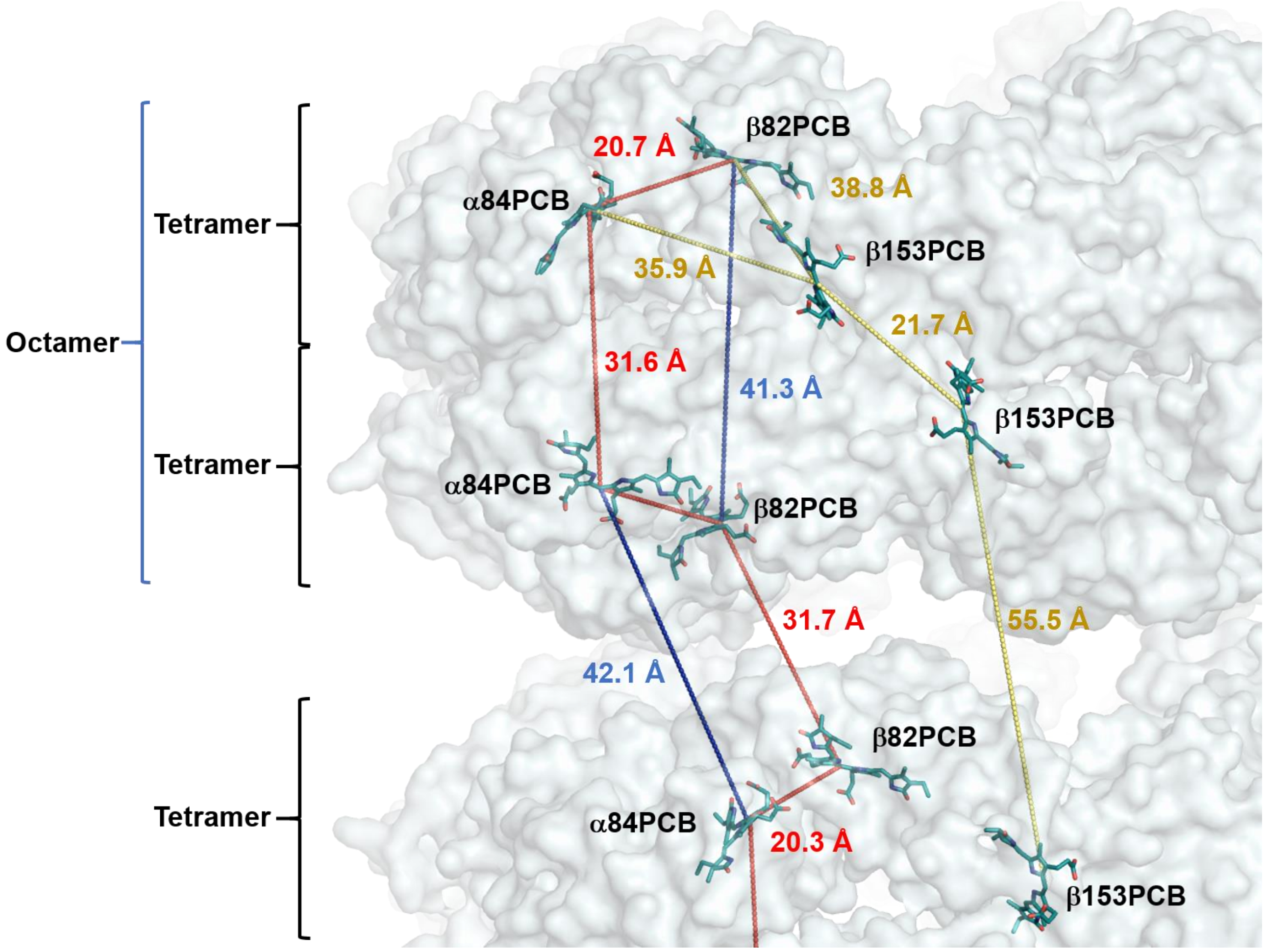

Supplementary Fig. 11 Intra- and inter-protein PCB distances of *Tl*CPC-8. PCBs in *Tl*CPC-8 are represented by cyan sticks. CPCs are shown in cyan surface representation with 70% transparency. The proposed energy transfer pathway (the shortest distances between PCBs) is shown in red. The next shortest distances are colored in blue. Distances involved in β153PCB are colored in yellow.

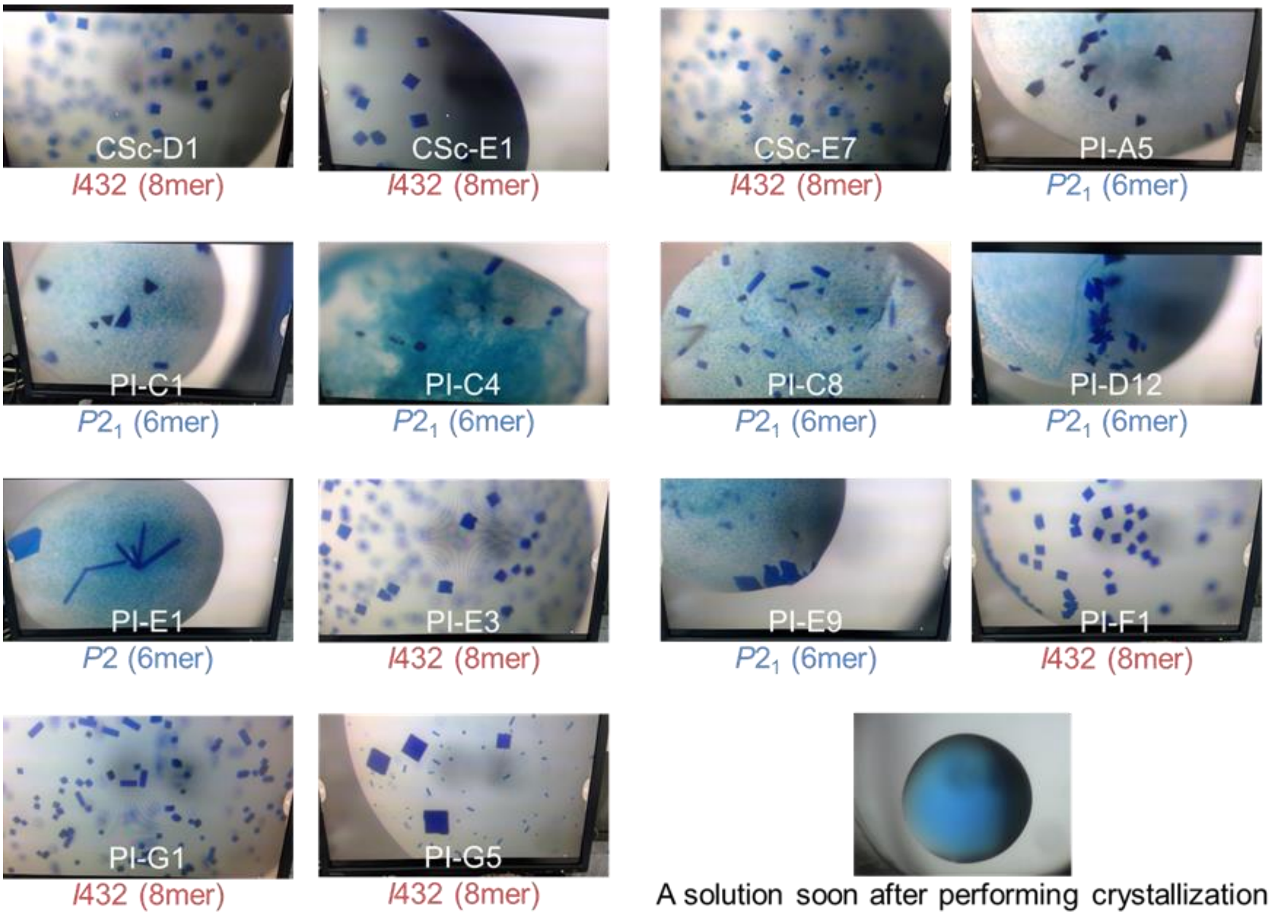

Supplementary Fig. 12 Photos of crystals. Representative photos of *Tl*CPC crystals. Crystallizations were performed using the commercially available screens from Hampton Research. PI = PEG/Ion Screen^{TM}, CSc = Crystal Screen Cryo^{TM}. See Supplementary Table 1 for the detail conditions.

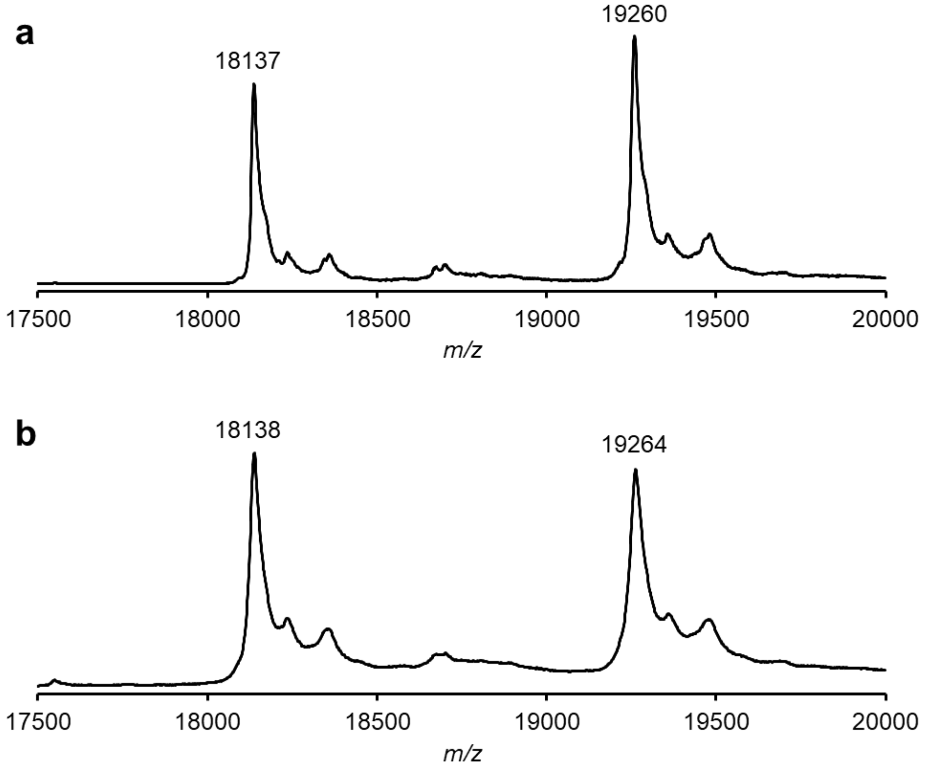

Supplementary Fig. 13 MALDI-TOF mass spectra. a Solution of single crystals of *Tl*CPC-6 and **b** solution of single crystals of *Tl*CPC-8. The signals at *m/z* 18137 and 18138 were assignable to the α subunit of *Tl*CPC with a PCB (calcd. 18130.29), and the signal at *m/z* 19260 and 19264 were assignable to the β subunit of *Tl*CPC with two PCBs and one methylated βAsn72 (calcd. 19249.87).

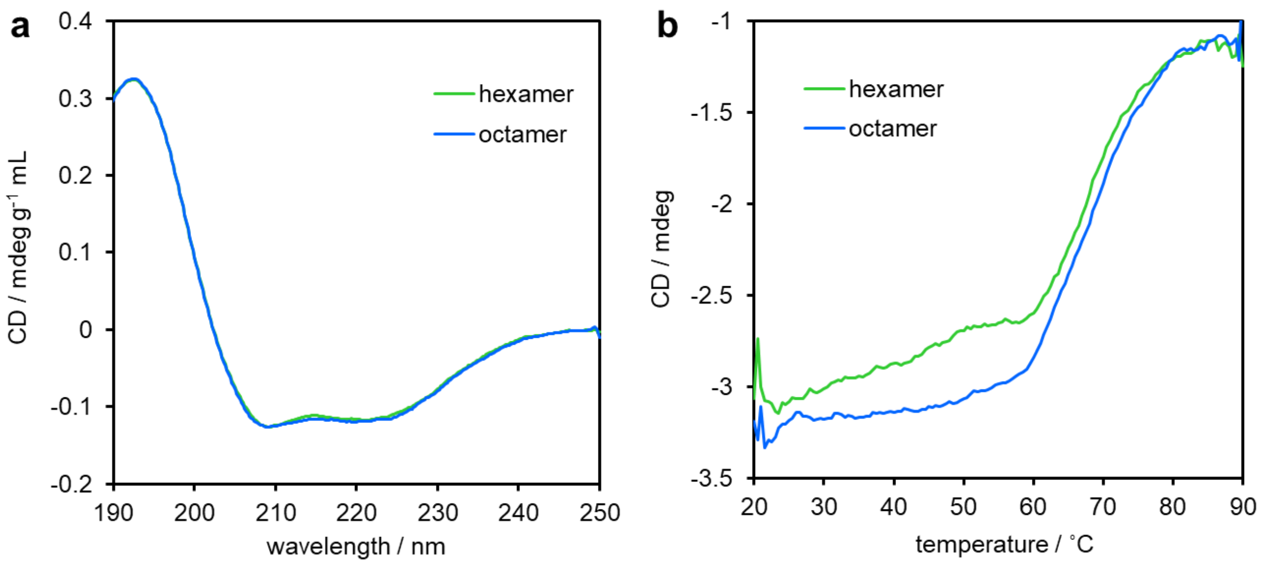

Supplementary Fig. 14 CD spectra of CPC solutions of single crystals. a CD spectra of CPC solutions prepared by dissolving single crystals of *Tl*CPC-6 or *Tl*CPC-8 into 10 mM potassium phosphate buffer (pH 7.0). Note that the concentrations of these solutions (0.057 mg/mL for hexamer, 0.047 mg/mL for octamer), which were determined by measuring UV-vis spectra, were low enough to disassemble into monomers. **b** Temperature dependences of CD at 222 nm. The concentrations of the solutions were as follows; 0.034 mg/mL for hexamer, 0.027 mg/mL for octamer. The thermal denaturation midpoints were calculated as 71°C for both solutions.

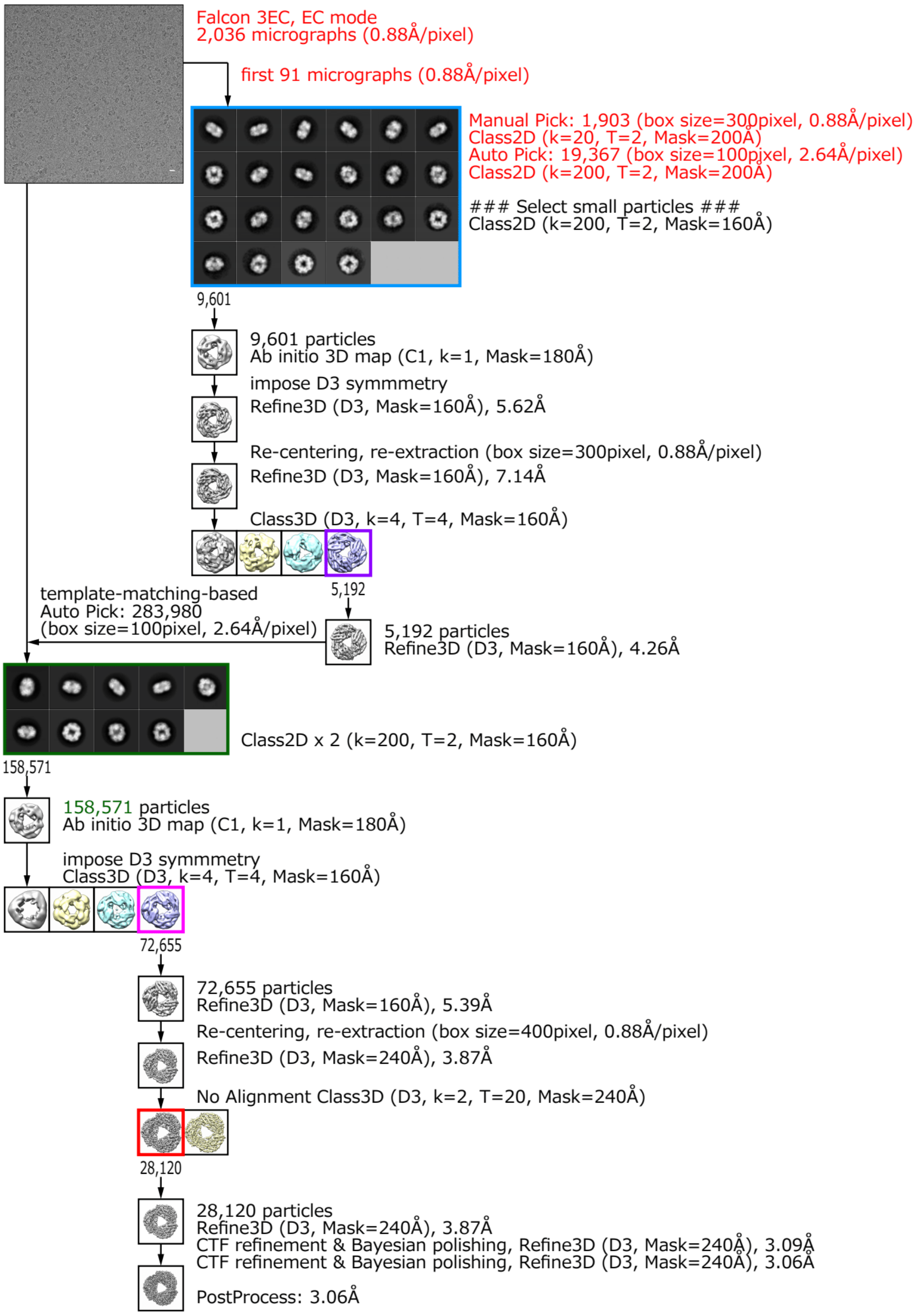

Supplementary Fig. 15 Cryo-EM data processing for *Tl*CPC-6. Data processing workflow used to obtain the final cryo-EM map of *Tl*CPC-6. The particle images of *Tl*CPC-6 and *Tl*CPC-8 are extracted from the same micrographs, and thus, the processing steps with red texts are the same as *Tl*CPC-8 (Supplementary Fig. 17). Please see the "Details of the cryo-EM data processing in Supplementary Fig. 15–18" section in the Supporting information for details.

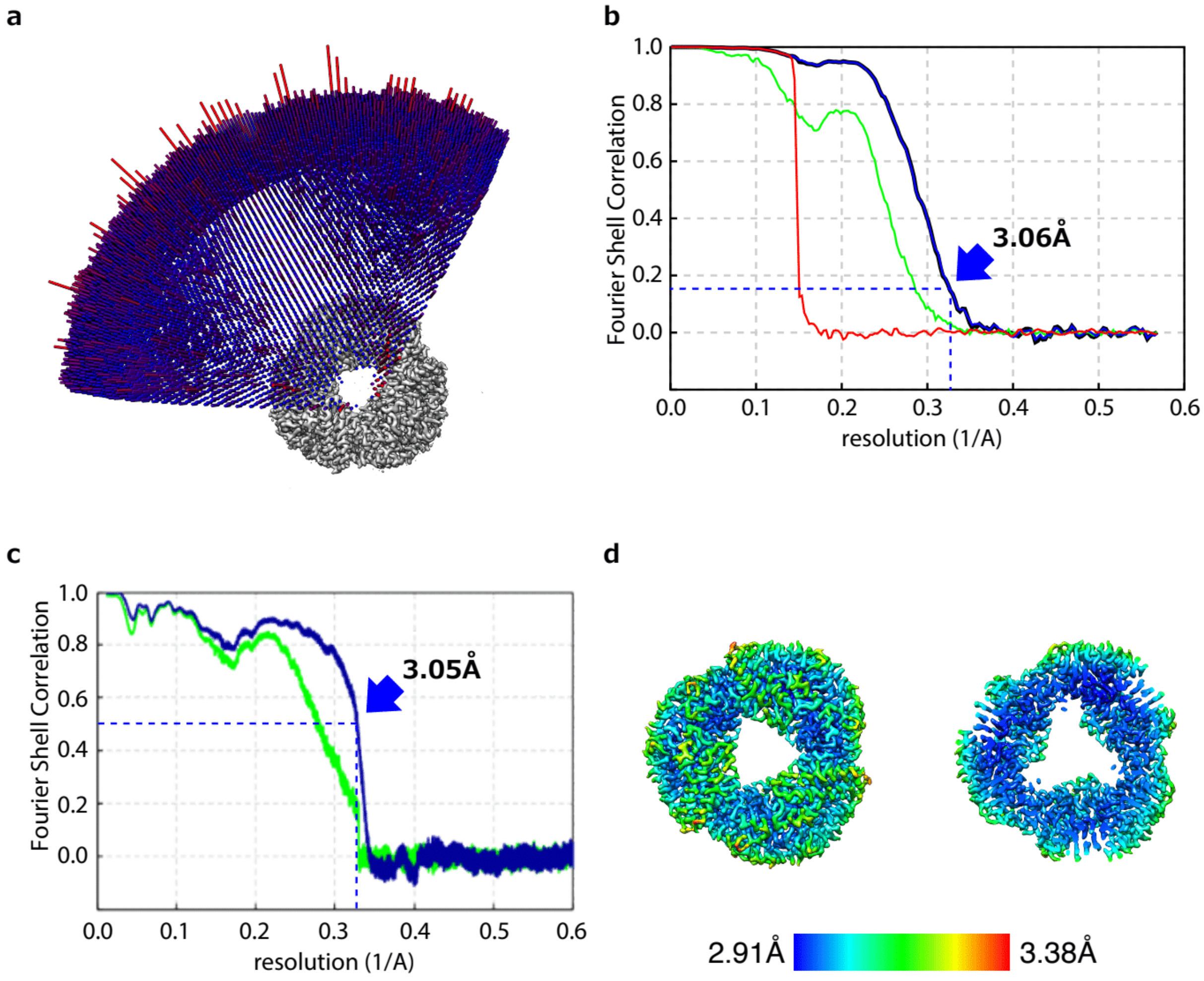

Supplementary Fig. 16 Cryo-EM data processing for *Tl*CPC-6. a Orientation distribution, **b** half-sets FSC curves, **c** map-to-model FSC curve, and **d** outer (left) and inner (right) local resolutions of the cryo-EM maps of *Tl*CPC-6. In the half-sets FSC curves, FSC corrected (black), FSC unmasked (green), FSC masked (blue), and corrected FSC phase randomized (red) maps are shown.

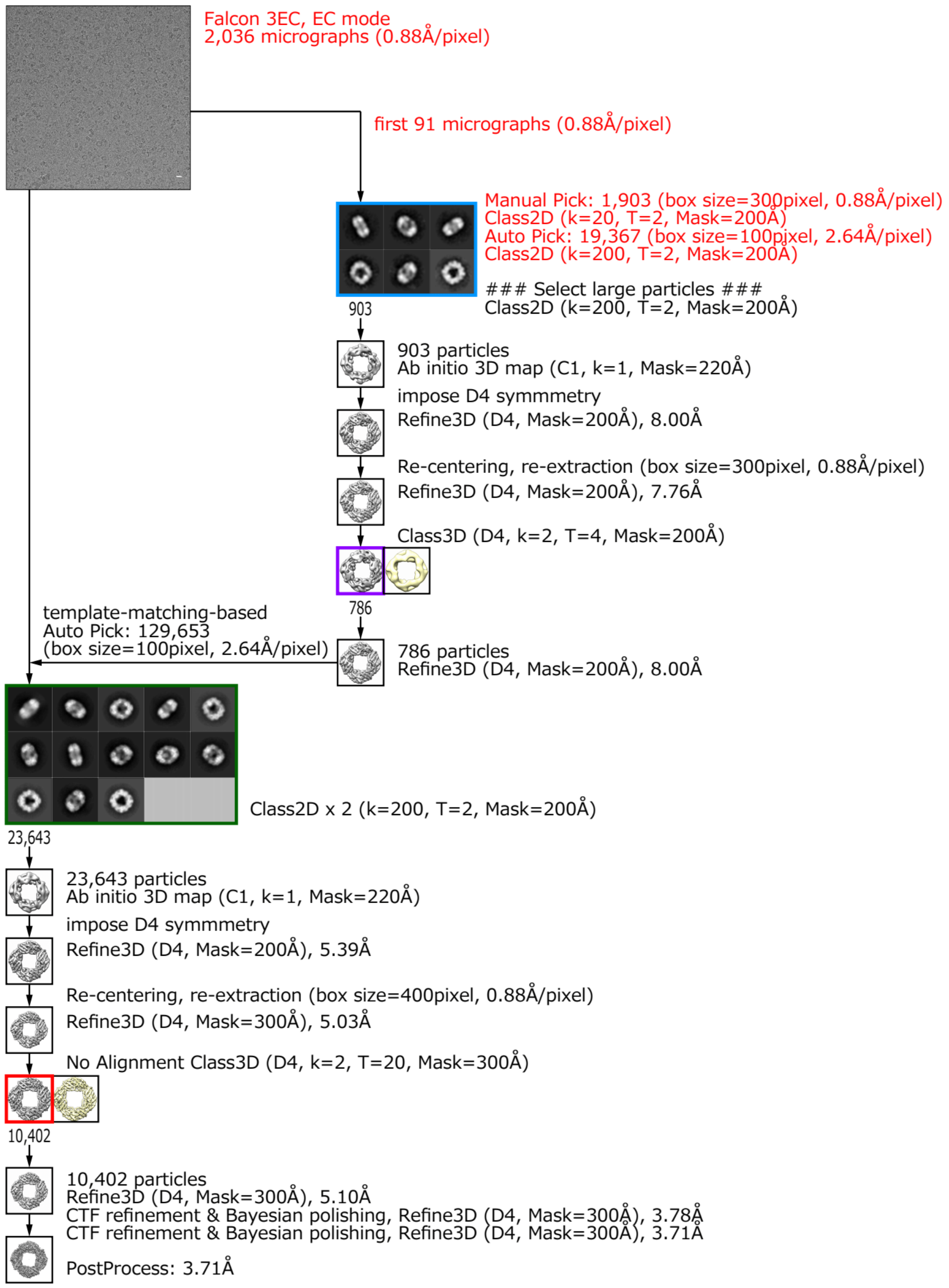

Supplementary Fig. 17 Cryo-EM data processing for *Tl*CPC-8. Data processing workflow used to obtain the final cryo-EM map of *Tl*CPC-8. The particle images of *Tl*CPC-6 and *Tl*CPC-8 are extracted from the same micrographs, and thus, the processing steps with red texts are the same as *Tl*CPC-6 (Supplementary Fig. 15). Please see the "Details of the cryo-EM data processing in Supplementary Fig. 15–18" section in the Supporting information for details.

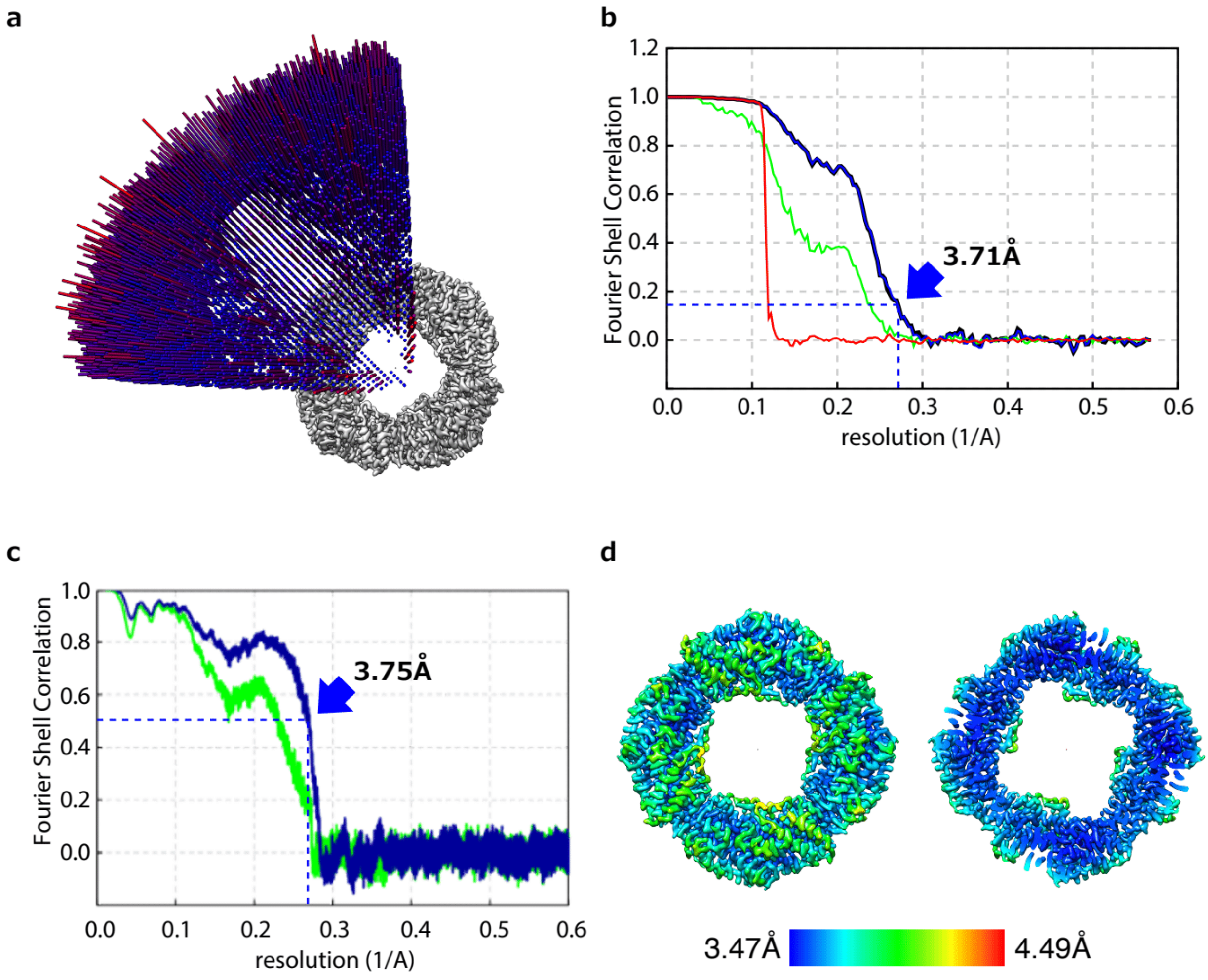

Supplementary Fig. 18 Cryo-EM data processing for *Tl*CPC-8. a Orientation distribution, **b** half-sets FSC curves, **c** map-to-model FSC curve, and **d** outer (left) and inner (right) local resolutions of the cryo-EM maps of *Tl*CPC-8. In the half-sets FSC curves, FSC corrected (black), FSC unmasked (green), FSC masked (blue), and corrected FSC phase randomized (red) maps are shown.

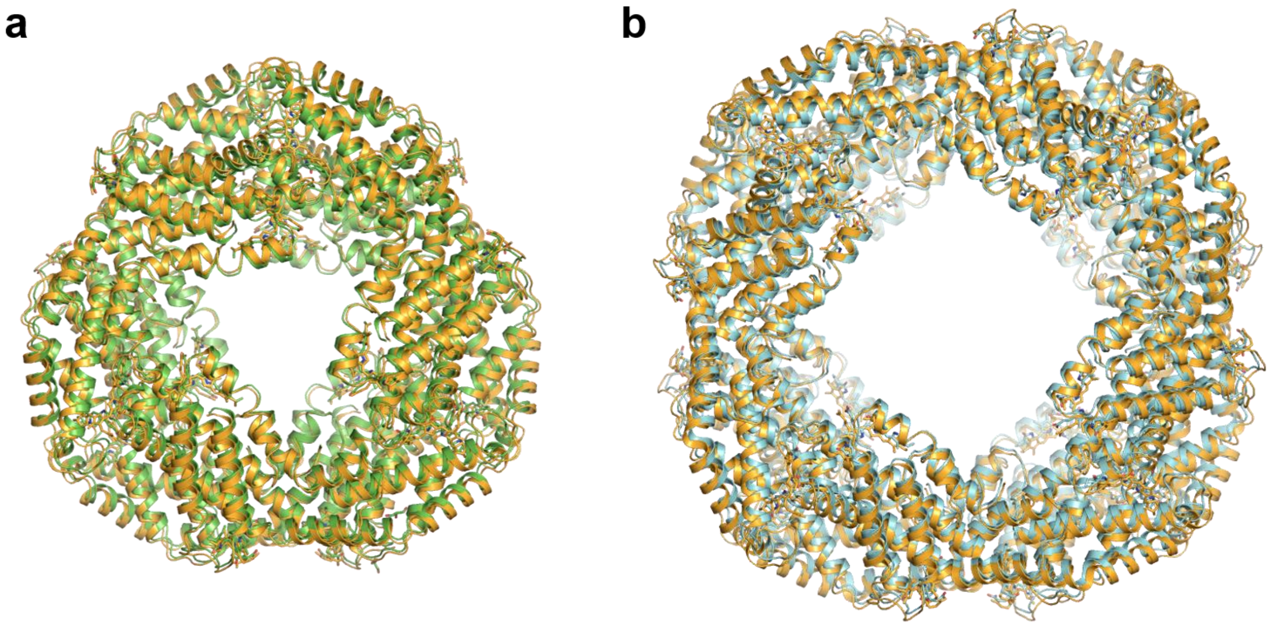

Supplementary Fig. 19 Superimposition of crystal and cryo-EM models of *Tl*CPC. The superimposition of crystal and cryo-EM models of *Tl*CPC-6 (**a**) and *Tl*CPC-8 (**b**). Crystal (green and cyan) and cryo-EM (orange) models are represented by ribbon models.

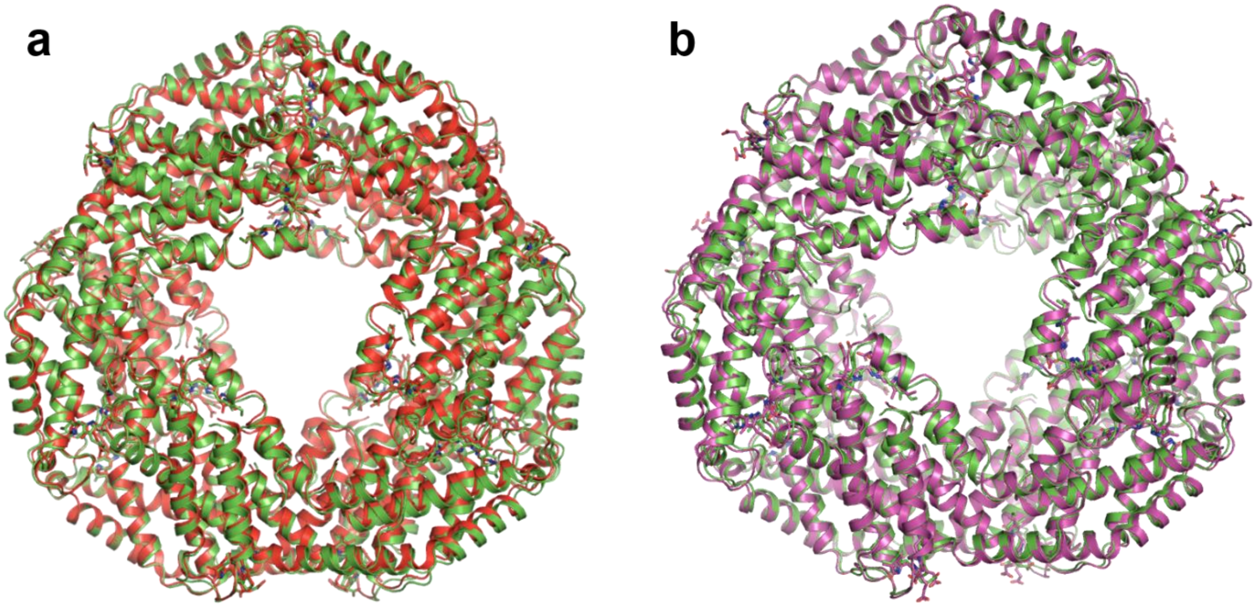

Supplementary Fig. 20 Superimposition of cryo-EM models of *Tl*CPC and PCs in PBS. a,b The superimpositions of cryo-EM models of *Tl*CPC-6 (green), *Gp*PC (red), and *Pp*PC (purple).

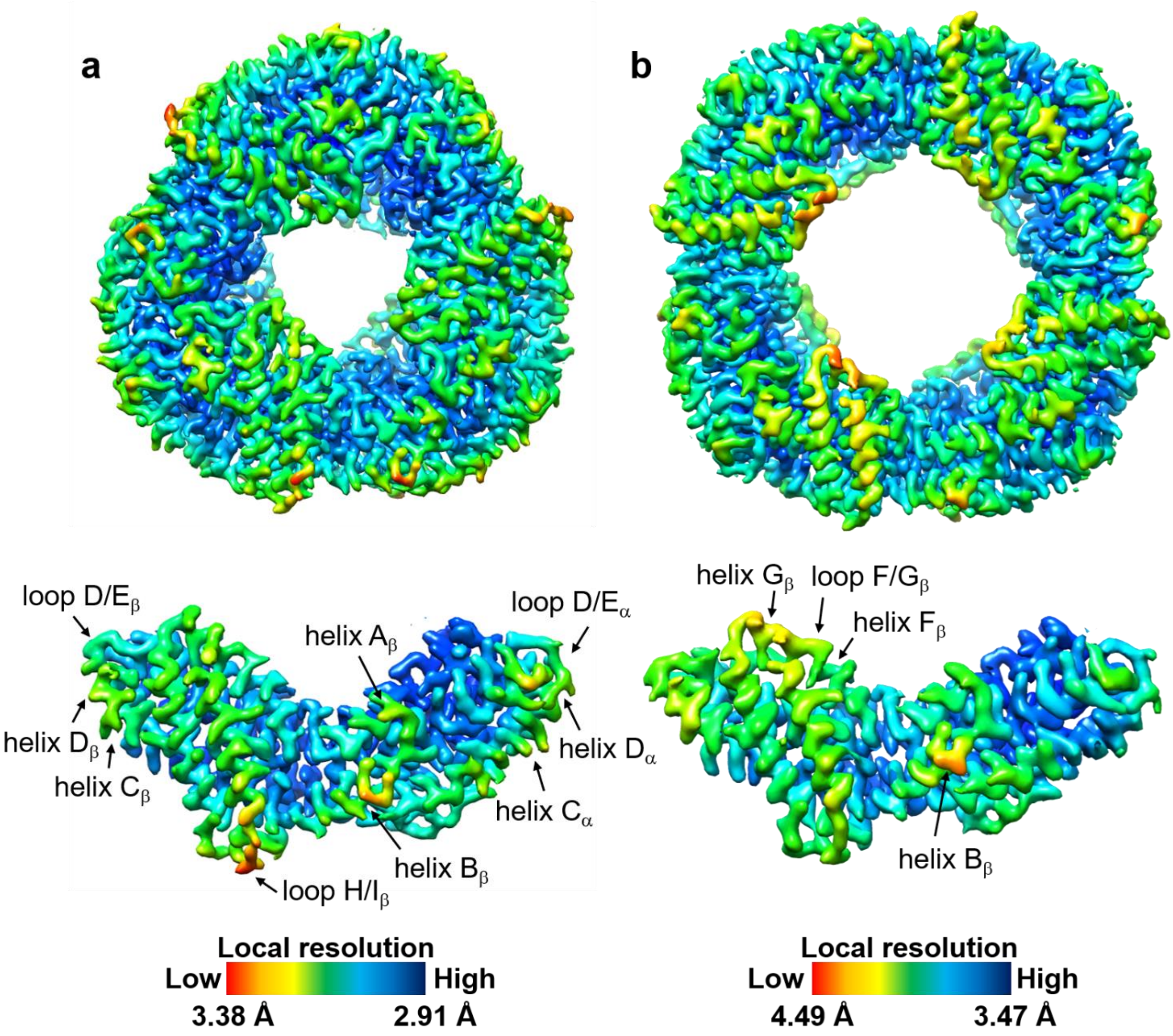

Supplementary Fig. 21 Local resolution maps of *Tl*CPC. The cryo-EM local resolution maps of *Tl*CPC-6 (**a**) and *Tl*CPC-8 (**b**). The models are colored according to local resolutions. The corresponding local resolution values are indicated by color-bars.

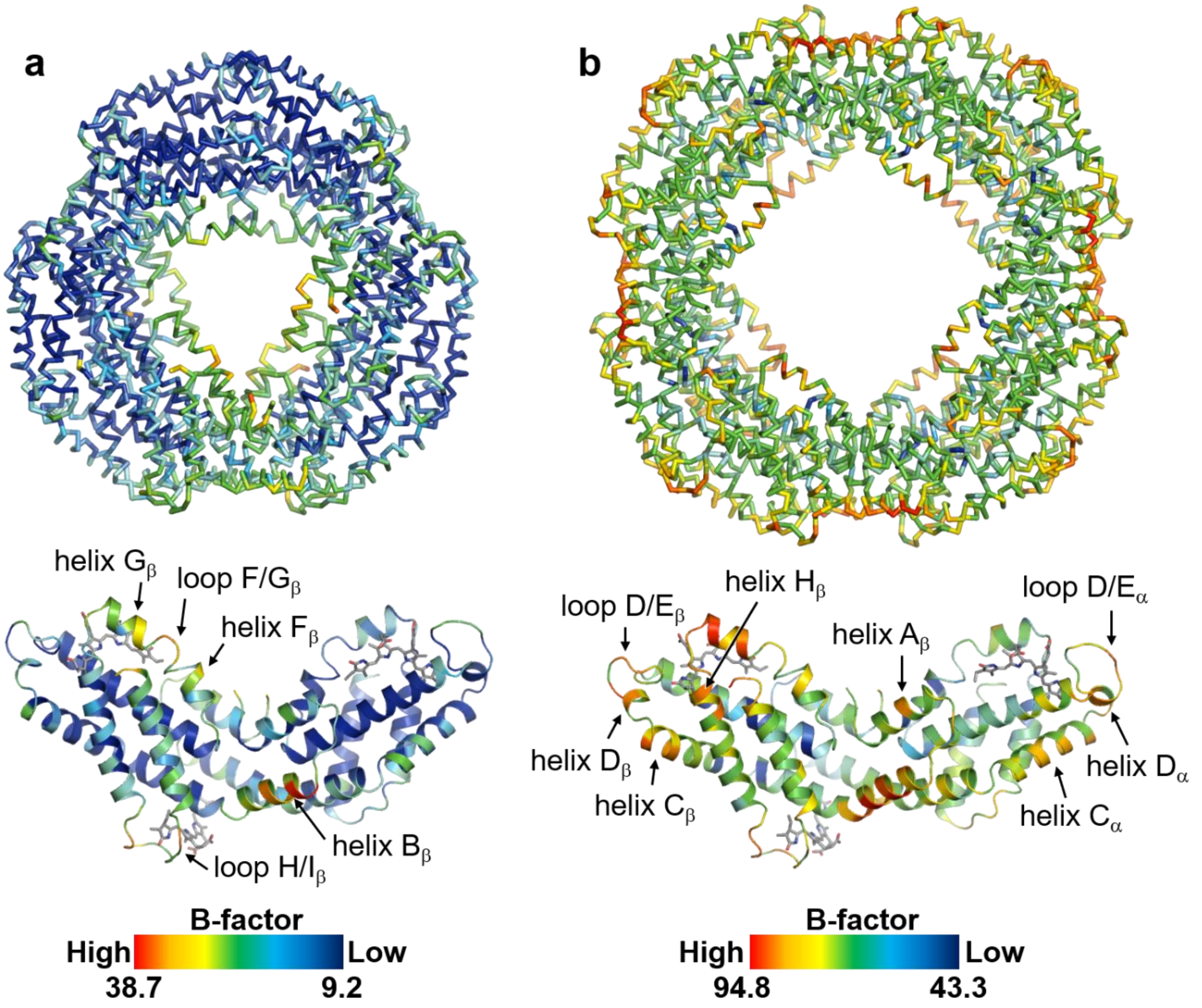

Supplementary Fig. 22 B-factor distributions of *Tl*CPC. Crystal models of *Tl*CPC-6 (**a**) and *Tl*CPC-8 (**b**). The models are colored according to B factors (blue to red spectra depicts increasing B-factor values). B-factor of Cα atom represents B-factor of each residues.

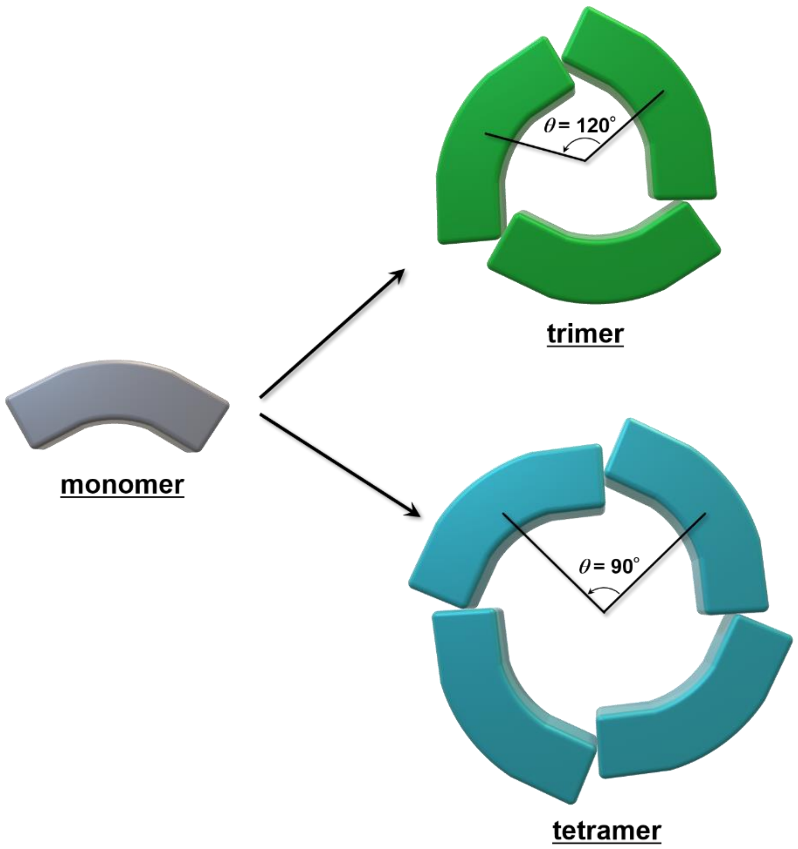

Supplementary Fig. 23 Formation of closed circular oligomers from monomers. Formation of trimer and tetramer from the same monomeric structures, where the rotation angles (θ) of trimer and tetramer are 120° and 90°, respectively, resulted in $\Delta\theta$ of 30°. Monomeric structures are represented by bended cuboids.

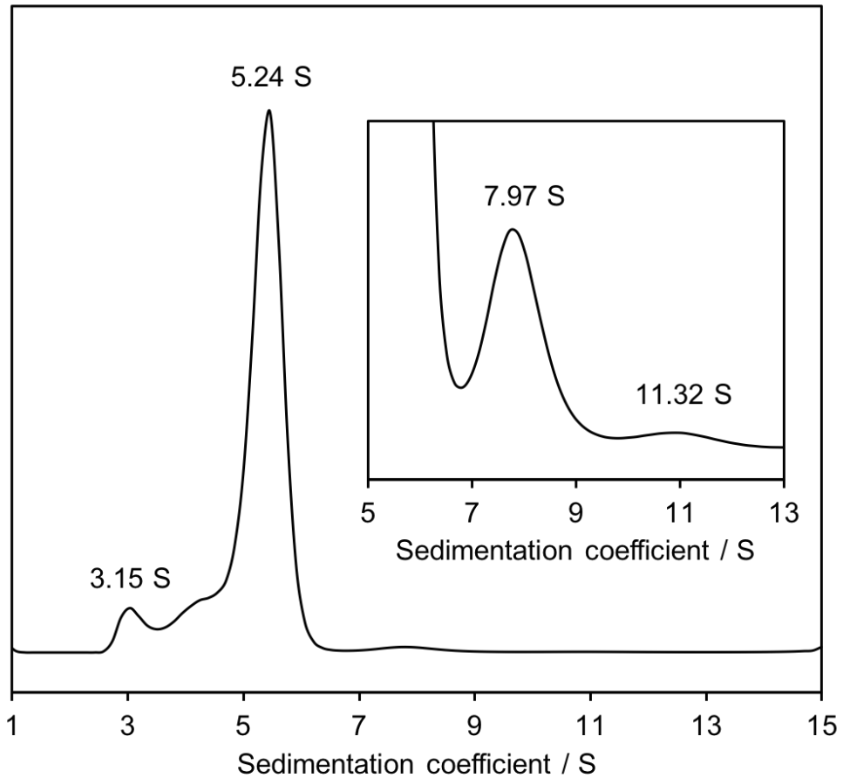

Supplementary Fig. 24 Sedimentation coefficient distribution of *Tl*CPC. Sedimentation velocity analytical ultracentrifugation of *Tl*CPC (0.92 mg/mL) in 10 mM potassium phosphate buffer solution (pH 7.0) was performed. The data was analyzed by SEDFIT to obtain the sedimentation coefficient distribution. Inset: enlarged view of the distribution in the range of 5–13 S.

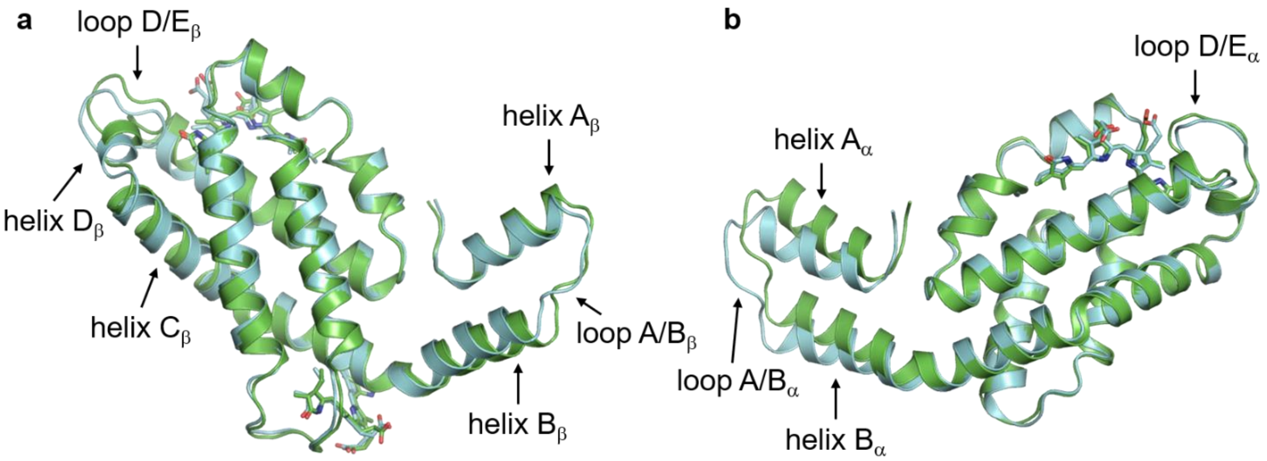

Supplementary Fig. 25 Superimposition of monomers. The superimposition of crystal models of **a** β- and **b** α-subunits in *Tl*CPC-6 (green) and *Tl*CPC-8 (cyan) represented by ribbon models.

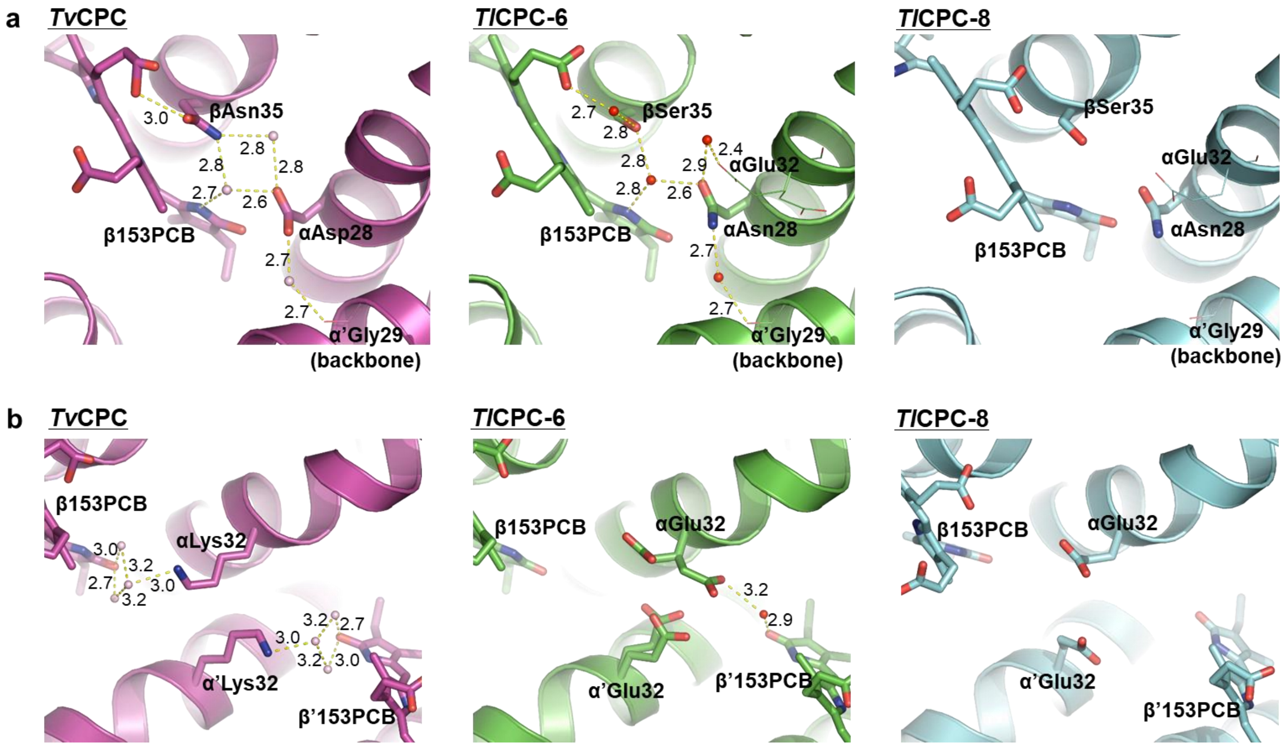

Supplementary Fig. 26 Hydrogen bond networks for CPC assembly. a Key hydrogen bond networks to form hexameric structure in *Tv*CPC. **b** Hydrogen bonds in the dimeric interface. *Tv*CPC (magenta), *Tl*CPC-6 (green), and *Tl*CPC-8 (cyan) represented by ribbon models. Water molecules are represented by pink or red spheres, and hydrogen bond networks are represented by yellow dashed lines with corresponding hydrogen bond distances (Å). Note that water molecules were not assigned in *Tl*CPC-8 due to the low resolution.

a b

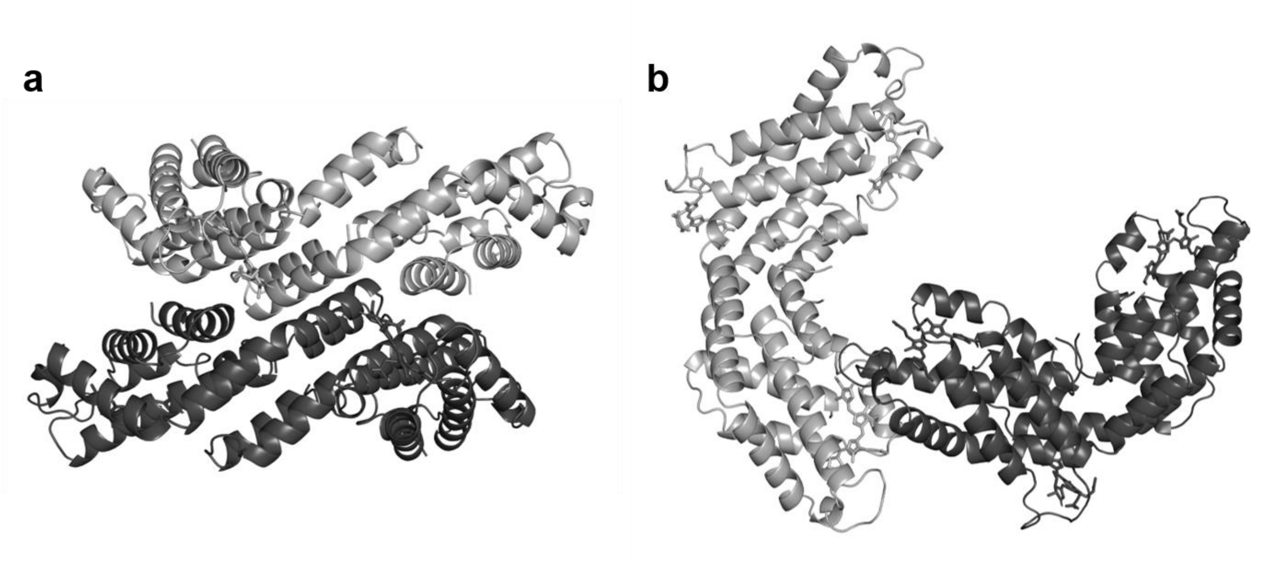

Supplementary Fig. 27 Possible dimeric structures of CPC. a One-third of a hexamer and **b** two-thirds of a trimer, where the contact% are 26% and 4%, respectively. Monomeric units are represented by black and grey ribbon models.

Supplementary Table 1 Summary of the crystallization conditions of *Tl*CPC.

Entry	Space group	*a* (Å)	*b* (Å)	*c* (Å)	*α* (°)	*β* (°)	*γ* (°)	State	Crystallization conditions[a]				
1	$P2_1$ (No. 4)	60.5	188.1	106.9	90.0	92.6	90.0	6mer	PI	A3	0.2 M NH_4F	pH 6.2	20% PEG3350
2	$P2_1$ (No. 4)	60.6	188.1	106.9	90.0	92.9	90.0	6mer	PI	A5	0.2 M $MgCl_2$	pH 5.9	20% PEG3350
3	$P2_1$ (No. 4)	60.7	188.6	109.4	90.0	99.9	90.0	6mer	PI	A12	0.2 M NH_4I	pH 6.2	20% PEG3350
4	$P2_1$ (No. 4)	60.7	187.8	106.8	90.0	92.9	90.0	6mer	PI	C1	0.2 M $Mg(OAc)_2$	pH 7.9	20% PEG3350
5	$P2_12_12$ (No. 18)	107.7	119.8	188.5	90.0	90.0	90.0	6mer	PI	C3	0.2 M NaOAc	pH 8.0	20% PEG3350
6	$P2_1$ (No. 4)	60.6	188.0	107.0	90.0	92.9	90.0	6mer	PI	C4	0.2 M $Ca(OAc)_2$	pH 7.5	20% PEG3350
7	$P2_1$ (No. 4)	60.6	188.1	106.4	90.0	92.5	90.0	6mer	PI	C8	0.2 M $MgSO_4$	pH 6.0	20% PEG3350
8	$P2_12_12$ (No. 18)	60.3	187.6	210.4	90.0	90.0	90.0	6mer	PI	D5	0.2 M KH_2PO_4	pH 4.8	20% PEG3350
9	$P2_12_12$ (No. 18)	60.2	187.6	210.1	90.0	90.0	90.0	6mer	PI	D5	0.2 M KH_2PO_4	pH 4.8	20% PEG3350
10	$P2_1$ (No. 4)	81.8	211.4	138.4	90.0	103.4	90.0	6mer	PI	D12	0.2 M NH_4 Citrate	pH 5.1	20% PEG3350
11	$P2_12_12$ (No. 18)	60.2	187.5	210.4	90.0	90.0	90.0	6mer	PI	D12	0.2 M NH_4 Citrate	pH 5.1	20% PEG3350
12	$P2$ (No. 2)	92.9	111.6	146.9	90.0	108.2	90.0	6mer	PI	E1	0.1 M Na Malonate	pH 4.0	12% PEG3350
13	$P2_1$ (No. 4)	82.3	212.1	138.8	90.0	103.5	90.0	6mer	PI	E9	4% Tacsimate	pH 4.0	12% PEG3350
14	$P2_12_12$ (No. 18)	60.1	187.4	210.1	90.0	90.0	90.0	6mer	PI	E12	8% Tacsimate	pH 5.0	20% PEG3350
15	$P2_1$ (No. 4)	60.6	188.1	213.6	90.0	92.9	90.0	6mer	PI	E12	8% Tacsimate	pH 5.0	20% PEG3350
16	$P2_1$ (No. 4)	60.6	188.1	106.8	90.0	92.9	90.0	6mer	PI	F6	8% Tacsimate	pH 8.0	20% PEG3350
17	$P2_1$ (No. 4)	60.4	187.9	106.7	90.0	92.6	90.0	6mer	PI	F6	8% Tacsimate	pH 8.0	20% PEG3350
18	$P2$ (No. 2)	60.4	187.7	106.9	90.0	92.7	90.0	6mer	PI	F12	0.2 M DL-Malic acid	pH 7.0	20% PEG3350
19	$P2_12_12$ (No. 18)	107.7	115.1	187.0	90.0	90.0	90.0	6mer	PI	G4	0.2 M HCOONa	pH 7.0	20% PEG3350
20	$P2_1$ (No. 4)	60.6	188.0	107.9	90.0	94.0	90.0	6mer	PI	G4	0.2 M HCOONa	pH 7.0	20% PEG3350
21	$P2$ (No. 2)	80.9	103.7	138.8	90.0	104.4	90.0	6mer	PI	G7	2% Tacsimate, 0.1 M NaOAc	pH 4.0	16% PEG3350
22	$P2$ (No. 2)	81.8	105.5	136.8	90.0	103.3	90.0	6mer	PI	G7	2% Tacsimate, 0.1 M NaOAc	pH 4.0	16% PEG3350
23	$P2_12_12$ (No. 18)	108.4	115.9	188.4	90.0	90.0	90.0	6mer	PI	H3	50 mM Citric acid, 50 mM Bis-Tris propane	pH 5.0	16% PEG3350
24	$P2_1$ (No. 4)	60.9	188.2	107.8	90.0	94.1	90.0	6mer	PI	H3	50 mM Citric acid, 50 mM Bis-Tris propane	pH 5.0	16% PEG3350
25	$P2_1$ (No. 4)	60.4	188.1	106.9	90.0	92.7	90.0	6mer	Ind	F12	0.2 M NaCl, 0.1 M HEPES	pH 7.5	25% PEG3350
26	$P2_1$ (No. 4)	62.3	193.9	110.4	90.0	94.0	90.0	6mer	Ind	C6	0.1 M NaCl, 0.1 M Bis-Tris	pH 6.5	1.5 M NH_4SO_4
27	$I432$ (No. 211)	231.1	231.1	231.1	90.0	90.0	90.0	8mer	PI	E3	0.1 M Na Malonate	pH 5.0	12% PEG3350
28	$I432$ (No. 211)	230.9	230.9	230.9	90.0	90.0	90.0	8mer	PI	F1	4% Tacsimate	pH 6.0	12% PEG3350
29	$I432$ (No. 211)	230.8	230.8	230.8	90.0	90.0	90.0	8mer	PI	F11	0.1 M DL-Malic acid	pH 7.0	12% PEG3350
30	$I432$ (No. 211)	232.4	232.4	232.4	90.0	90.0	90.0	8mer	PI	G1	0.1 M NaOAc	pH 7.0	12% PEG3350
31	$I432$ (No. 211)	233.2	233.2	233.2	90.0	90.0	90.0	8mer	PI	G5	0.1 M NH_4 Tartrate	pH 7.0	12% PEG3350

Supplementary Table 1 (continued).

Entry	Space group	*a* (Å)	*b* (Å)	*c* (Å)	α (°)	β (°)	γ (°)	State	Crystallization conditions[a]					
32	*I*432 (No. 211)	230.0	230.0	230.0	90.0	90.0	90.0	8mer	CSc	A1	20 mM $CaCl_2$, 0.1 M NaCl	pH 4.6	30% MPD	
33	*I*432 (No. 211)	229.5	229.5	229.5	90.0	90.0	90.0	8mer	CSc	A7	70 mM Na_2CO_3	pH 6.5	0.98 M NaOAc	30% Glycerol
34	*I*432 (No. 211)	230.2	230.2	230.2	90.0	90.0	90.0	8mer	CSc	B2	0.19 M $CaCl_2$, 95 mM HEPES	pH 7.5	26.6% PEG400	5% Glycerol
35	*I*432 (No. 211)	228.7	228.7	228.7	90.0	90.0	90.0	8mer	CSc	C8			1.5 M NH_4SO_4	25% Glycerol
36	*I*432 (No. 211)	230.4	230.4	230.4	90.0	90.0	90.0	8mer	CSc	E1	1.6 M NaCl		8% PEG6000	20% Glycerol
37	*I*432 (No. 211)	229.7	229.7	229.7	90.0	90.0	90.0	8mer	CSc	F2	0.15 M K/Na Tartrate, 75 mM Na Citrate	pH 5.6	1.5 M NH_4SO_4	25% Glycerol
38	*I*432 (No. 211)	229.9	229.9	229.9	90.0	90.0	90.0	8mer	CSc	G10	37.5 mM $CdSO_4$, 75 mM HEPES	pH 7.5	0.75 M NaOAc	25% Glycerol
39	*I*432 (No. 211)	229.5	229.5	229.5	90.0	90.0	90.0	8mer	CSc	H1	75 mM HEPES	pH 7.5	7.5% PEG8000, 6% EG	25% Glycerol

[a] Crystallizations were performed using the commercially available screens from Hampton Research. PI = PEG/Ion Screen™, Ind = Index™, CSc = Crystal Screen Cryo™, Bis-Tris propane = 1,3-Bis[tris(hydroxymethyl)methylamino]propane, HEPES = 4-(2-hydroxyethyl)-1-piperazineethanesulfonic acid, MPD = 2-methyl-2,4-pentanediol, PEG = polyethylene glycol, EG = ethylene glycol.

Supplementary Note 1 Details of the cryo-EM data processing in Supplementary Fig. 15–18.

First, the movie fractions were aligned, dose-weighted, and averaged using MotionCor2 on 5 × 5 tiled fractions with a B-factor of 300[S1]. The non-weighted movie sums were used for Contrast Transfer Function (CTF) estimation with the Gctf program[S2]. The dose-weighted sums were used for all subsequent steps of image processing. The subsequent processes of particle picking, two-dimensional (2D) classification, *ab initio* reconstruction, three-dimensional (3D) classification, 3D refinement, CTF refinement, and Bayesian polishing were performed using RELION-3.0[S3]. Initially, 1,903 particles were manually picked and performed 2D classification for preparing a 2D reference of template-matching-based auto-pick by RELION-3. From the first 91 micrographs, 19,367 particles were automatically picked and extracted while rescaling to 2.64 Å/pixel with 100-pixel box size. The extracted particle images were subjected to the second reference-free 2D classification (200 expected classes, 200 Å mask diameter). The resultants show small and large particle images, which were used for the subsequent analyses of *Tl*CPC-6 and *Tl*CPC-8, respectively.

For *Tl*CPC-6, the 9,601 particles corresponding to the best 22 classes, which had around 100 Å diameter and displayed secondary-structural elements, were selected from the result of the second reference-free 2D classification, and then used for *ab initio* reconstruction (asymmetry, single expected class, 180 Å mask diameter). D_3 symmetry was imposed on the generated *ab initio* map, which was used as an initial 3D reference for the 3D refinements (D_3 symmetry, 160 Å mask diameter). The refined volume and particle images were rescaled to 0.88 Å/pixel with a 300-pixel box size and used for the 3D refinement (D_3 symmetry, 160 Å mask diameter). The generated 3D refined map was used for 3D classification (4 expected classes, 160 Å mask diameter). The 3D volume and 5,192 particles of the best 3D class, which displayed the highest resolution, were used for the subsequent 3D refinements (D_3 symmetry, 160 Å mask diameter).

The generated 3D refined map was used as a 3D reference for template-matching-based auto-pick and a stack of 283,980 particle images was extracted from the 2,036 dose-weighted sum micrographs while rescaling to 2.64 Å/pixel with 100-pixel box size. The images were subjected to two consecutive runs of reference-free 2D classification (200 expected classes, 160 Å mask diameter). Next, the 158,571 particles corresponding to the best 9 classes, which displayed secondary-structural elements, were selected for *ab initio* reconstruction (asymmetry, single expected class, 180 Å mask diameter).

*D*3 symmetry was imposed on the generated *ab initio* map, which was used as an initial 3D reference for the 3D classification with *D*3 symmetry (4 expected classes, 160 Å mask diameter). The 3D volume and 72,655 particles of the best 3D class, which displayed the highest resolution, were used as an initial 3D reference for the 3D refinements (*D*3 symmetry, 160 Å mask diameter). The generated 3D refined map was rescaled to 0.88 Å/pixel with a 400-pixel box size and used for the subsequent 3D refinements (*D*3 symmetry, 240 Å mask diameter). The 3D refined map was used for no-alignment 3D classification with *D*3 symmetry (2 expected classes, 240 Å mask diameter). The 3D volume and 28,120 particles of the best 3D class, which displayed the highest resolution, were used for the subsequent 3D refinements (*D*3 symmetry, 240 Å mask diameter). The cycle of CTF refinement and Bayesian polishing was repeated two times. The 3D refinement (*D*3 symmetry, 240 Å mask diameter) with a soft-edged 3D mask (15-pixel extension, 30-pixel soft cosine edge) was executed after each Bayesian polishing step. The last 3D refinement (*D*3 symmetry, 240 Å mask diameter) with a soft-edged 3D mask (15-pixel extension, 30-pixel soft cosine edge) and post-processing generated the final result at 3.06 Å resolution.

For *Tl*CPC-8, the 903 particles corresponding to the best 6 classes, which had around 130 Å diameter and displayed secondary-structural elements, were selected from the result of the second reference-free 2D classification, and then used for *ab initio* reconstruction (asymmetry, single expected class, 220 Å mask diameter). *D*4 symmetry was imposed on the generated *ab initio* map, which was used as an initial 3D reference for the 3D refinements (*D*4 symmetry, 200 Å mask diameter). The refined volume and particle images were rescaled to 0.88 Å/pixel with a 300-pixel box size and used for the 3D refinement (*D*4 symmetry, 200 Å mask diameter). The generated 3D refined map was used for 3D classification (2 expected classes, 200 Å mask diameter). The 3D volume and 786 particles of the best 3D class, which displayed the highest resolution, were used for the subsequent 3D refinements (*D*4 symmetry, 200 Å mask diameter).

The generated 3D refined map was used as a 3D reference for template-matching-based auto-pick and a stack of 129,653 particle images was extracted from the 2,036 dose-weighted sum micrographs while rescaling to 2.64 Å/pixel with 100-pixel box size. The images were subjected to two consecutive runs of reference-free 2D classification (200 expected classes, 200 Å mask diameter). Next, the 23,643 particles corresponding to the best 13 classes, which displayed secondary-structural elements, were selected for *ab initio* reconstruction (asymmetry, single expected class, 220 Å mask diameter). *D*4

symmetry was imposed on the generated *ab initio* map, which was used as an initial 3D reference for the 3D refinements (D_4 symmetry, 200 Å mask diameter). The generated 3D refined map was rescaled to 0.88 Å/pixel with a 400-pixel box size and used for the subsequent 3D refinements (D_4 symmetry, 300 Å mask diameter). The 3D refined map was used for no-alignment 3D classification with D4 symmetry (2 expected classes, 300 Å mask diameter). The 3D volume and 10,402 particles of the best 3D class, which displayed the highest resolution, were used for the subsequent 3D refinements (D_4 symmetry, 300 Å mask diameter). The cycle of CTF refinement and Bayesian polishing was repeated two times. The 3D refinement (D_4 symmetry, 300 Å mask diameter) with a soft-edged 3D mask (15-pixel extension, 30-pixel soft cosine edge) was executed after each Bayesian polishing step. The last 3D refinement (D_4 symmetry, 300 Å mask diameter) with a soft-edged 3D mask (15-pixel extension, 30-pixel soft cosine edge) and post-processing generated the final result at 3.71 Å resolution.

For calculation of the global resolution estimation after each 3D refinement, the gold-standard Fourier Shell Correlation (FSC) resolution with a criterion of 0.143 was used[S4]. The local resolution was estimated using the implementation of RELION-3. The model-to-map FSC resolution with 0.5 criterion was calculated using phenix.mtriage[S5]. For the visualization of the output 3D images, UCSF Chimera was used[S6].

Supplementary references

[S1] Zheng, S. Q. *et al.* MotionCor2: anisotropic correction of beam-induced motion for improved cryo-electron microscopy. *Nat. Methods* **14**, 331–332 (2017).

[S2] Zhang, K. Gctf: Real-time CTF determination and correction. *J. Struct. Biol.* **193**, 1–12 (2016).

[S3] Zivanov, J. *et al.* New tools for automated high-resolution cryo-EM structure determination in RELION-3. *eLife* **7**, e42166 (2018).

[S4] Rosenthal, P. B. & Henderson, R. Optimal determination of particle orientation, absolute hand, and contrast loss in single-particle electron cryomicroscopy. *J. Mol. Biol.* **333**, 721–745 (2003).

[S5] Afonine, P. V. *et al.* New tools for the analysis and validation of cryo-EM maps and atomic models. *Acta Crystallogr. D Struct. Biol.* **74**, 814–840 (2018).

[S6] Pettersen, E. F. *et al.* UCSF Chimera–a visualization system for exploratory research and analysis. *J. Comput. Chem.* **25**, 1605–1612 (2004).
